# Analysis of Cybersecurities within Industrial Control Systems Using Interval-Valued Complex Spherical Fuzzy Information

**DOI:** 10.1155/2022/3304333

**Published:** 2022-02-22

**Authors:** Abdulrahman Alothaim, Shoukat Hussain, Suheer Al-Hadhrami

**Affiliations:** ^1^STC's Artificial Intelligence Chair, Department of Information Systems, College of Computer and Information Sciences, King Saud University, Riyadh 11543, Saudi Arabia; ^2^Department of Mathematics, Institute of Numerical Sciences, Gomal University, Dera Ismail Khan 29050, KPK, Pakistan; ^3^Computer Engineering Department, Engineering College, Hadhramout University, Hadhramout, Al Mukalla, Yemen

## Abstract

Technology affects almost every aspect of life and is constantly changing. Digital communication technology has made it easier and faster to connect people all over the world. Digital technology is used in varies fields, including business, industries, companies, and educational institutions. There are various benefits of technology; it is also associated with a number of risks and dangerous threats known as cybercrimes. Cybercrime is a criminal activity that targets digital technology, like a computer, a computer network, or a mobile device. Cybersecurity is the way we reduce the risk of becoming a victim of cybercrime. Cybersecurity is the process of defending against cyberattacks. By using these concepts, we investigated the interval-valued complex T-spherical fuzzy relations (IVCT-spherical-FRs) introduced in this paper. We studied the relationships between different types of cybersecurity and the sources of cyberattacks. Furthermore, the Hasse diagram for the interval-valued complex T-spherical partial order set and relation is developed. The concepts of Hasse diagram are being used to examine various cybersecurity techniques and practices. The most effective method is identified using the features of Hasse diagrams. Finally, comparison tests are used to demonstrate the benefits of the proposed methods.

## 1. Introduction

Uncertainty is a part of human life, resulting from a variety of aspects ranging from a lack of conviction to unawareness. The uncertainty caused by randomness is measured by probability. Uncertainty interferes with our capability to prepare for the future and is produced by confusing or imprecise data. Zadeh [1] was the first to introduce the concept of fuzzy sets (FSs) in 1965. An FS is defined by a mapping with the unit interval [0, 1] as its range. A degree of membership is the name given to this mapping. The FS theory of Zadeh is used to model imperfect information and uncertainty. Klir [2] invented the crisp relations, which are used to examine the relationships between crisp sets. However, scholars have long struggled to model ambiguity and uncertainty. Mendel [3] established the relations for FS, which are known as fuzzy relations (FRs). Unlike classical relations, FRs are not limited to yes-or-no problems. They can indicate the level, quality, and degree of good or healthy relations among any pair of FSs based on their membership level. The greater values of degree of membership represent that the relation is a strong one; on the other hand, the lower values represent the weak relationship. FR is a larger idea than classical relationships, and it can discuss problems in both situations. An FR becomes a classical relation when the membership values are set to 0 and 1. Zadeh [4] proposed the concept of interval-valued FSs (IVFSs) in 1975. The IVFSs are an expansion of Zadeh's FSs. These sets are intervals that are a subset of the unit interval and define the grade of membership. It is impossible for an expert to describe his or her certainty with an exact real number; an interval that indicates the level of certainty is appropriate. As a result, the IVFSs support in simulating uncertainty as well as the implications of human ignorance, blunders, and confusion. Bustince and Burillo [5] devised the idea of interval-valued fuzzy relations (IVFRs), which expand classical relations and FRs. Deschrijver and Kerre [6] investigated the relationships between several fuzzy set theory expansions. Goguen [7] proposed an axiom system for a relative variant of FS theory. Zywica [8] used FSs to represent medical uncertainties. A comment on Zadeh's extensions was proposed by Roman-Flores et al. [9]. Gehrke et al. [10] conducted a review on IVFSs. Bustince [11] applied IVFSs to comparative reasoning.

Ramot et al. [12] proposed the novel concept of using complex valued mappings as the membership degrees of a set in 2002. They proposed the complex fuzzy set (CFS), where the degree of membership is determined by values from a complex plane's unit disc. Therefore, the degrees of membership in a CFS are complex numbers; they are divided into two parts: real and imaginary. Each of these parts represents a distinct entity. The real part is said to be the amplitude term of the membership degree, although the imaginary part is said to be the phase term of the membership degree. CFSs are commonly used to model phase shifting problems. Furthermore, Ramot et al. [13] developed complex fuzzy relations (CFRs), which are utilized to find the relationships among CFSs. Nasir et al. [14] gave the IVCFRs and applied them in medical diagnosis. Greenfield et al. [15] introduced the concept of an interval-valued complex fuzzy set (IVCFS) by changing the degree of membership of a CFS from a single number to an interval. The CFSs were reviewed by Yazdanbakhsh and Dick [16]. Chen et al. [17] studied a neurofuzzy architecture by employing CFSs. Tamir et al. [18] presented some CFS applications; Dai et al. [19] constructed distance measures between IVCFSs; Greenfield et al. [20] defined the join and meet procedures for IVCFSs.

Although it was considered that there are many instances where FS cannot be implemented due to its limitations, Atanassov [21] discovered that an FS can become stronger if the level of nonmembership is added in its structure. As a result, he developed the intuitionistic FS (IFS). According to Atanassov, the values allocated to membership and nonmembership levels must be in the unit interval [0, 1], and the sum of both values should be within 0 and 1. The difference between the structure of an IFS and the structure of an FS is that an IFS discusses both the level of satisfaction represented by the level of membership and the level of discontent represented by the level of nonmembership. Burillo et al. [22] suggested the concept of intuitionistic FR (IFR), which investigates the relationships between any two IFSs. Li [23] utilized IFSs for multiattribute decision-making (DM) models and theories. Atanassov [24] introduced a new notion called the interval-valued intuitionistic fuzzy set (IVIFS) by expressing the degree of membership and nonmembership of IFS in the form of intervals. Alkouri et al. [25] evaluated the effect of complex numbers in IFSs and established the concept of complex intuitionistic fuzzy sets (CIFSs). A CIFS gives each of its elements a pair of functions whose values are complex numbers from a unit circle, as long as their total is also in the complex plane. Comparable to CFSs, the membership and nonmembership grades of CIFSs also consist of amplitude terms and phase terms. The interval-valued complex intuitionistic fuzzy relation (IVCIFR) was proposed by Garg and Rani [26]. IFSs were used in medical diagnosis by De et al. [27], pattern recognition was used by Vlachos and Sergiadis [28], and a comparison of IVFSs, IFSs, and bipolar-valued FSs was used by Lee et al. [29]. For calculating the distances between IVFSs and IFSs, Grzegorzewski [30] utilized the Hausdorff metric. Jan et al. [31] carried out research on cybersecurity and cybercrime in the oil and gas industries utilizing the novel structure of complex intuitionistic fuzzy relation (CIFR). Complex intuitionistic fuzzy classes were investigated by Ali et al. [32]. Liu and Jiang [33] introduced a new distance measure of IVIFSs and applied it in decision-making. The correlation of IVIFSs was developed by Bustince and Burillo [34].

Later, Cuong [35] proposed the picture FSs (PFSs) by including a degree of abstinence in the IFS structure. The levels of membership, abstinence, and nonmembership in a PFS all accept values from the unit interval as long as their sum is between 0 and 1. The correlation coefficients for picture fuzzy sets were first established by Singh et al. [36]. The new procedures of picture fuzzy relations and fuzzy comprehensive assessment were presented by Bo et al. [37]. The new operations on interval-valued picture fuzzy set, interval-valued picture fuzzy soft set, and their applications were described by Khalil et al. [38]. The idea of a decision-making model under a complex picture fuzzy set was introduced by Akram et al. [39]. Mahmood et al. [40] looked out an approach toward decision-making and medical diagnosis problems using the concept of spherical fuzzy sets. Similarity measures for T-spherical fuzzy sets were defined by Ullah et al. [41], with applications in pattern recognition. Ullah et al. [42] proposed employing interval-valued T-spherical fuzzy aggregation operators to evaluate investment policies based on multiattribute decision-making. Nasir et al. [43] proposed the concept of complex T-spherical fuzzy relations with their applications in economic relationships and international trades.

In this paper, the interval-valued complex T-spherical fuzzy sets (IVCT-spherical-FSs) and the novel concept of interval-valued complex T-spherical fuzzy relations (IVCT-spherical-FR) are discussed. The IVCT-spherical-FSs are more reliable than FSs, IFSs, CFSs, CIFSs, IVIFSs, and IVCIFSs. Testing IVCT-spherical-FSs on the proposed applications is simple because they contain complex-valued membership grades, abstinence grades, and nonmembership grades. The IVCT-spherical-FSs are well capable of dealing with the condition, as the addition of the grades remains within its limitations. Thus, IVCT-spherical-FSs cover all the previous methods and techniques that actually demonstrate the superiority of the concept of IVC-spherical-FSs and also a Cartesian product between two IVCT-spherical-FSs is investigated. We can now determine the associations between any two IVCT-spherical-FSs due to the implementation of IVCT-spherical-FRs. Furthermore, several examples, theorems, and definitions are used to study the different types of IVCT-spherical-FSs. The types include interval-valued complex T-spherical reflexive fuzzy relation (IVCT-spherical reflexive-FR), IVCT-spherical irreflexive-FR, IVCT-spherical symmetric-FR, IVCT-spherical antisymmetric-FR, IVCT-spherical asymmetric-FR, IVCT-spherical transitive-FR, IVCT-spherical composite-FR, IVCT-spherical equivalence-FR, IVCT-spherical preorder-FR, IVCT-spherical partial order-FR, IVCT-spherical complete-FR, IVCT-spherical linear order-FR, IVCT-spherical strict order-FR, converse of a IVCT-spherical-FR, and the equivalence classes for IVCT-spherical equivalence-FR. Hence, the IVCT-spherical-FR is a tool that finds out and analyzes the relationship between the IVCT-spherical-FSs.

In comparison to a crisp value membership grade, an interval-valued membership rating contains the decision-maker's blunders, miscommunications, and mistakes. Furthermore, complex-valued grades make modelling, difficulties of periodic nature, and phase transitions easier. This article examines the impact of computer technology on a wide range of disciplines, including business, industry, and educational institutions. These technological systems are extremely advantageous, although they are also subject to a variety of cybercrime-related attacks and risks. Cybercrime is tackled using a variety of techniques, practices, technologies, and methods known as cybersecurity. It might be difficult to determine the type of cybercrime at times, leaving various uncertainties. Similarly, because there are so many solutions available to combat these threats and risks, there are reservations about using the correct cybersecurity strategies to save the business or enterprises from attacks. Due to uncertainty and an inability to make the best decision, selecting the most appropriate security solution may be challenging. As a result, we employed fuzzy theory to overcome all of these uncertainties. In an industrial control system, this article mathematically explores the relationships between cybersecurity and the sources of cyberattacks, such as the effectiveness, neutral, and ineffectiveness of cybersecurity against a particular source. Importantly, the current study proposes a method for determining several types of cybersecurity and selecting the ideal one for a company or network. Hasse diagrams and IVCT-spherical partial order-FRs are the foundations of this novel method.

The following is a summary of the research: Section 1 contains the introduction and literature review.  Section 2 analyzes some of the predefined variables that are used in this research.  Section 3 discusses the interval-valued complex T-spherical fuzzy relations (IVCT-spherical-FRs) and their types along with examples and theorems. In Section 4, IVCT-spherical-FSs and IVCT-spherical-FRs have two applications. In the first application, cybercrime, cybersecurity, and penetration sources in system of industrial control are investigated. The second application uses Hasse diagrams and IVCT-spherical partial order-FRs to discover the most effective cybersecurity strategy. In Section 5, in the discipline of fuzzy set theory, present structures are compared to suggested structures. Finally, the paper ends with the conclusion in Section 6.

## 2. Preliminaries

This section contains all the necessary definitions and discussions of CFSs, IVCFSs, CIFS, IVCIFS, CPFS, C-spherical-FSs, and CT-spherical-FSs.

**Definition 1 (see [12]).**

Let *H* be a universal set; a set *E* is called complex fuzzy set (CFS) if

(1) $$\begin{array}{c} E=\left\{x,\psi_{E}\left(x\right)e^{\left(\xi_{E}\left(x\right)\right)2\pi \iota }\right\}, \end{array}$$

where *ψ*_*E*_(*x*), *ξ*_*E*_(*x*) :  *H*⟶[0,1] are called the amplitude and phase terms of the degree of membership, respectively.

**Definition 2 (see [15]).**

Let *H* be a universal set; a set *E* is called interval-valued complex fuzzy set (IVCFS) if

(2) $$\begin{array}{c} E=\left\{x,\left[\psi_{E}^{-}\left(x\right),\psi_{E}^{+}\left(x\right)\right]e^{\left[\xi_{E}^{-}\left(x\right),\xi_{E}^{+}\left(x\right)\right]2\pi \iota }\right\}, \end{array}$$

where [*ψ*_*E*_^−^(*x*), *ψ*_*E*_^+^(*x*)] :  *H*⟶[0,1] is called the amplitude term of membership grade, and [*ξ*_*E*_^−^(*X*), *ξ*_*E*_^+^(*x*)] :  *H*⟶[0,1] is called the amplitude term of membership grade.

**Definition 3 (see [25]).**

Let *H* be a universal set; a set *E* is called complex intuitionistic fuzzy set (CIFS) if

(3) $$\begin{array}{c} E=\left\{x,\psi_{\left(E\right)m}\left(x\right)e^{\left(\xi_{\left(E\right)m}\left(x\right)\right)2\pi \iota },\psi_{\left(E\right)n}\left(x\right)e^{\left(\xi_{\left(E\right)n}\left(x\right)\right)2\pi \iota }\right\}, \end{array}$$

where *ψ*_(*E*)*m*_(*x*), *ψ*_(*E*)*n*_(*x*) :  *H*⟶[0,1] are called the amplitude terms of the degrees of membership and nonmembership, respectively, and *ξ*_(*E*)*m*_(*x*), *ξ*_(*E*)*n*_(*x*) : *H*⟶[0,1] are called the phase terms of the degrees of membership and nonmembership, respectively.

**Definition 4 (see [26]).**

Let *H* be a universal set; a set *E* is called interval-valued complex intuitionistic fuzzy set (IVCIFS) if

(4) $$\begin{array}{c} E=\left\{\left(x\;,\left[\psi_{\left(E\right)m}^{-}\left(x\right),\psi_{\left(E\right)m}^{+}\left(x\right)\right]e^{\left[\xi_{\left(E\right)m}^{-}\left(x\right),\xi_{\left(E\right)m}^{+}\left(x\right)\right]2\pi \iota },\left[\psi_{\left(E\right)n}^{-}\left(x\right),\psi_{\left(E\right)n}^{+}\left(x\right)\right]e^{\left[\xi_{\left(E\right)n}^{-}\left(x\right),\xi_{\left(E\right)n}^{+}\left(x\right)\right]2\pi \iota }\right)\right\}, \end{array}$$

where the mappings [*ψ*_(*E*)*m*_^−^(*x*), *ψ*_(*E*)*m*_^+^(*x*)], [*ψ*_(*E*)*n*_^−^(*x*), *ψ*_(*E*)*n*_^+^(*x*)] : *H*⟶[0,1] are called the amplitude terms of membership and nonmembership grades, respectively, and [*ξ*_(*E*)*m*_^−^(*x*), *ξ*_(*E*)*m*_^+^(*x*)], [*ξ*_(*E*)*n*_^−^(*x*), *ξ*_(*E*)*n*_^+^(*x*)] : *H*⟶[0,1] are called the phase terms of membership and nonmembership grades, respectively.

**Definition 5 (see [39]).**

Let *H* be a universal set; a set *E* is called complex picture fuzzy set (CPFS) if

(5) $$\begin{array}{c} E=\left\{x,\psi_{\left(E\right)m}\left(x\right)e^{\left(\xi_{\left(E\right)m}\left(x\right)\right)2\pi \iota },\psi_{\left(E\right)a}\left(x\right)e^{\left(\xi_{\left(E\right)a}\left(x\right)\right)2\pi \iota },\psi_{\left(E\right)n}\left(x\right)e^{\left(\xi_{\left(E\right)n}\left(x\right)\right)2\pi \iota }\right\}, \end{array}$$

where the mappings *ψ*_(*E*)*m*_(*x*), *ψ*_(*E*)*a*_(*x*), *ψ*_(*E*)*n*_(*x*) :  *H*⟶[0,1] are called the amplitude terms of membership, abstinence, and nonmembership, respectively, and *ξ*_(*E*)*m*_(*x*), *ξ*_(*E*)*a*_(*x*), *ξ*_(*E*)*n*_(*x*) : *H*⟶[0,1] are called the phase terms of membership, abstinence, and nonmembership, respectively, which satisfy the following condition:

(6) $$\begin{array}{c} 0\leq \left(\psi_{\left(E\right)m}^{+}\left(x\right)\right)+\left(\psi_{\left(E\right)a}\left(x\right)\right)+\left(\psi_{\left(E\right)n}^{+}\left(x\right)\right)\leq 1\text{ and},\\ 0\leq \left(\xi_{\left(E\right)m}^{+}\left(x\right)\right)+\left(\xi_{\left(E\right)a}\left(x\right)\right)+\left(\xi_{\left(E\right)n}^{+}\left(x\right)\right)\leq 1\text{ where }n\in Z^{+}. \end{array}$$

**Definition 6 (see [43]).**

Let *H* be a universal set; a set *E* is called complex spherical fuzzy set (C-spherical-FS) if

(7) $$\begin{array}{c} E=\left\{x,\psi_{\left(E\right)m}\left(x\right)e^{\left(\xi_{\left(E\right)m}\left(x\right)\right)2\pi \iota },\psi_{\left(E\right)a}\left(x\right)e^{\left(\xi_{\left(E\right)a}\left(x\right)\right)2\pi \iota },\psi_{\left(E\right)n}\left(x\right)e^{\left(\xi_{\left(E\right)n}\left(x\right)\right)2\pi \iota }\right\}, \end{array}$$

where the mappings *ψ*_(*E*)*m*_(*x*), *ψ*_(*E*)*a*_(*x*), *ψ*_(*E*)*n*_(*x*) : *H*⟶[0,1] are called the amplitude terms of membership, abstinence, and nonmembership, respectively, and *ξ*_(*E*)*m*_(*x*), *ξ*_(*E*)*a*_(*x*), *ξ*_(*E*)*n*_(*x*) : *H*⟶[0,1] are called the phase terms of membership, abstinence, and nonmembership, respectively, which satisfy the following condition:

(8) $$\begin{array}{c} 0\leq {\left(\psi_{\left(E\right)m}^{+}\left(x\right)\right)}^{2}+{\left(\psi_{\left(E\right)a}\left(x\right)\right)}^{2}+{\left(\psi_{\left(E\right)n}^{+}\left(x\right)\right)}^{2}\leq 1\text{ and},\\ 0\leq {\left(\xi_{\left(E\right)m}^{+}\left(x\right)\right)}^{2}+{\left(\xi_{\left(E\right)a}\left(x\right)\right)}^{2}+{\left(\xi_{\left(E\right)n}^{+}\left(x\right)\right)}^{2}\leq 1\text{ where }n\in Z^{+}. \end{array}$$

**Definition 7 (see [43]).**

Let *H* be a universal set; a set *E* is called complex T-spherical fuzzy set (CT-spherical-FS) if

(9) $$\begin{array}{c} E=\left\{x,\psi_{\left(E\right)m}\left(x\right)e^{\left(\xi_{\left(E\right)m}\left(x\right)\right)2\pi \iota },\psi_{\left(E\right)a}\left(x\right)e^{\left(\xi_{\left(E\right)a}\left(x\right)\right)2\pi \iota },\psi_{\left(E\right)n}\left(x\right)e^{\left(\xi_{\left(E\right)n}\left(x\right)\right)2\pi \iota }\right\}, \end{array}$$

where the mappings *ψ*_(*E*)*m*_(*x*), *ψ*_(*E*)*a*_(*x*), *ψ*_(*E*)*n*_(*x*) : *H*⟶[0,1] are called the amplitude terms of membership, abstinence, and nonmembership, respectively, and *ξ*_(*E*)*m*_(*x*), *ξ*_(*E*)*a*_(*x*), *ξ*_(*E*)*n*_(*x*) :  *H*⟶[0,1] are called the phase terms of membership, abstinence, and nonmembership, respectively, which satisfy the following condition:

(10) $$\begin{array}{c} 0\leq {\left(\psi_{\left(E\right)m}^{+}\left(x\right)\right)}^{n}+{\left(\psi_{\left(E\right)a}\left(x\right)\right)}^{n}+{\left(\psi_{\left(E\right)n}^{+}\left(x\right)\right)}^{n}\leq 1\text{ and},\\ 0\leq {\left(\xi_{\left(E\right)m}^{+}\left(x\right)\right)}^{n}+{\left(\xi_{\left(E\right)a}\left(x\right)\right)}^{n}+{\left(\xi_{\left(E\right)n}^{+}\left(x\right)\right)}^{n}\leq 1\text{ where }n\in Z^{+}. \end{array}$$

## 3. Main Results

This section introduces the novel concepts of an IVCT-spherical-FSs and Cartesian product of two IVCT-spherical-FSs, as well as an IVCT-spherical-FR and its subtypes. A relevant example is provided for each definition. Furthermore, several interesting IVCT-spherical-FR outcomes have been obtained.

**Definition 8 .**

Let *H* be a universal set; a set *E* is called interval-valued complex T-spherical fuzzy set (IVCT-spherical-FS) if

(11) $$\begin{array}{c} {\;}^{\prime \prime }E=\left\{\left(\begin{array}{c} x,\left[\psi_{\left({\;}^{\prime \prime }E\right)\overset{\prime }{m}}^{-}\left(x\right),\psi_{\left({\;}^{\prime \prime }E\right)\overset{\prime }{m}}^{+}\left(x\right)\right]e^{\left[\xi_{\left({\;}^{\prime \prime }E\right)\overset{\prime }{m}}^{-}\left(x\right),\xi_{\left({\;}^{\prime \prime }E\right)\overset{\prime }{m}}^{+}\left(x\right)\right]2\pi l}\\ x,\left[\psi_{\left({\;}^{\prime \prime }E\right)\overset{\prime }{\alpha }}^{-}\left(x\right),\psi_{\left({\;}^{\prime \prime }E\right)\overset{\prime }{\alpha }}^{+}\left(x\right)\right]e^{\left[\xi_{\left({\;}^{\prime \prime }E\right)\overset{\prime }{\alpha }}^{-}\left(x\right),\xi_{\left({\;}^{\prime \prime }E\right)\overset{\prime }{\alpha }}^{+}\left(x\right)\right]2\pi l}\\ x,\left[\psi_{\left({\;}^{\prime \prime }E\right)\overset{\prime }{n}}^{-}\left(x\right),\psi_{\left({\;}^{\prime \prime }E\right)\overset{\prime }{n}}^{+}\left(x\right)\right]e^{\left[\xi_{\left({\;}^{\prime \prime }E\right)\overset{\prime }{n}}^{-}\left(x\right),\xi_{\left({\;}^{\prime \prime }E\right)\overset{\prime }{n}}^{+}\left(x\right)\right]2\pi l} \end{array}\right):x\in H\right\}, \end{array}$$

where the mappings [*ψ*_(*E*)*m*_^−^(*x*), *ψ*_(*E*)*m*_^+^(*x*)], [*ψ*_(*E*)*a*_^−^(*x*), *ψ*_(*E*)*a*_^+^(*x*)], [*ψ*_(*E*)*n*_^−^(*x*), *ψ*_(*E*)*n*_^+^(*x*)] : *H*⟶[0,1] are called the amplitude terms of membership, abstinence, and nonmembership grades, respectively, and [*ξ*_(*E*)*m*_^−^(*x*), *ξ*_(*E*)*m*_^+^(*x*)], [*ξ*_(*E*)*a*_^−^(*x*), *ξ*_(*E*)*a*_^+^(*x*)], [*ξ*_(*E*)*n*_^−^(*x*), *ξ*_(*E*)*n*_^+^(*x*)] : *H*⟶[0,1] are called the phase terms of membership, abstinence, and nonmembership grades, respectively, on the condition that

(12) $$\begin{array}{c} 0\leq {\left(\psi_{\left(E\right)m}^{+}\left(x\right)\right)}^{n}+{\left(\psi_{\left(E\right)a}^{+}\left(x\right)\right)}^{n}+{\left(\psi_{\left(E\right)n}^{+}\left(x\right)\right)}^{n}\leq 1\text{ and,}\\ 0\leq {\left(\xi_{\left(E\right)m}^{+}\left(x\right)\right)}^{n}+{\left(\xi_{\left(E\right)a}^{+}\left(x\right)\right)}^{n}+{\left(\xi_{\left(E\right)n}^{+}\left(x\right)\right)}^{n}\leq 1\text{ where n}\in {\text{Z}}^{+}. \end{array}$$

**Definition 9 .**

Suppose that $E=\left\{\left(\begin{array}{c} \left(x,y\right)\left[\psi_{\left({\;}^{\prime \prime }E\right)\overset{\prime }{m}}^{-}\left(x,y\right),\psi_{\left({\;}^{\prime \prime }E\right)\overset{\prime }{m}}^{+}\left(x,y\right)\right]e^{\left[\xi_{\left({\;}^{\prime \prime }E\right)\overset{\prime }{m}}^{-}\left(x,y\right),\xi_{\left({\;}^{\prime \prime }E\right)\overset{\prime }{m}}^{+}\left(x,y\right)\right]2\pi l,}\\ \left(x,y\right)\left[\psi_{\left({\;}^{\prime \prime }E\right)\overset{\prime \prime }{\alpha }}^{-}\left(x,y\right),\psi_{\left({\;}^{\prime \prime }E\right)\overset{\prime \prime }{\alpha }}^{+}\left(x,y\right)\right]e^{\left[\xi_{\left({\;}^{\prime \prime }E\right)\overset{\prime \prime }{\alpha }}^{-}\left(x,y\right),\xi_{\left({\;}^{\prime \prime }E\right)\overset{\prime \prime }{\alpha }}^{+}\left(x,y\right)\right]2\pi l,}\\ \left(x,y\right),\left[\psi_{\left({\;}^{\prime \prime }E\right)\overset{\prime }{n}}^{-}\left(x,y\right),\psi_{\left({\;}^{\prime \prime }E\right)\overset{\prime }{n}}^{+}\left(x,y\right)\right]e^{\left[\xi_{\left({\;}^{\prime \prime }E\right)\overset{\prime }{n}}^{-}\left(x,y\right),\xi_{\left({\;}^{\prime \prime }E\right)\overset{\prime }{n}}^{+}\left(x,y\right)\right]2\pi l} \end{array}\right):x\in H\right\}$ and $F=\left\{\left(\begin{array}{c} x,\left[\psi_{\left(F\right)\overset{\prime }{m}}^{-}\left(x\right),\psi_{\left(F\right)\overset{\prime }{m}}^{+}\left(x\right)\right]e^{\left[\xi_{\left(F\right)\overset{\prime }{m}}^{-}\left(x\right),\xi_{\left(F\right)\overset{\prime }{m}}^{+}\left(x\right)\right]2\pi l}\\ x,\left[\psi_{\left(F\right)\overset{\prime }{\alpha }}^{-}\left(x\right),\psi_{\left(F\right)\overset{\prime }{\alpha }}^{+}\left(x\right)\right]e^{\left[\xi_{\left(F\right)\overset{\prime }{\alpha }}^{-}\left(x\right),\xi_{\left(F\right)\overset{\prime }{\alpha }}^{+}\left(x\right)\right]2\pi l}\\ x,\left[\psi_{\left(F\right)\overset{\prime }{n}}^{-}\left(x\right),\psi_{\left(F\right)\overset{\prime }{n}}^{+}\left(x\right)\right]e^{\left[\xi_{\left(F\right)\overset{\prime }{n}}^{-}\left(x\right),\xi_{\left(F\right)\overset{\prime }{n}}^{+}\left(x\right)\right]2\pi l} \end{array}\right):y\in H\right\}$ are two IVCT-spherical-FSs in a universe *H*; then their Cartesian product is given as

(13) $$\begin{array}{c} E\times F=\left\{\left(\begin{array}{c} \left(x,y\right)\left[\psi_{\left({\;}^{\prime \prime }E\times F\right)\overset{\prime }{m}}^{-}\left(x,y\right),\psi_{\left({\;}^{\prime \prime }E\times F\right)\overset{\prime }{m}}^{+}\left(x,y\right)\right]e^{\left[\xi_{\left({\;}^{\prime \prime }E\times F\right)\overset{\prime }{m}}^{-}\left(x,y\right),\xi_{\left({\;}^{\prime \prime }E\times F\right)\overset{\prime }{m}}^{+}\left(x,y\right)\right]2\pi l,}\\ \left(x,y\right)\left[\psi_{\left({\;}^{\prime \prime }E\times F\right)\overset{\prime }{\alpha }}^{-}\left(x,y\right),\psi_{\left({\;}^{\prime \prime }E\times F\right)\overset{\prime }{\alpha }}^{+}\left(x,y\right)\right]e^{\left[\xi_{\left({\;}^{\prime \prime }E\times F\right)\overset{\prime }{\alpha }}^{-}\left(x,y\right),\xi_{\left({\;}^{\prime \prime }E\times F\right)\overset{\prime }{\alpha }}^{+}\left(x,y\right)\right]2\pi l,}\\ \left(x,y\right)\left[\psi_{\left({\;}^{\prime \prime }E\times F\right)\overset{\prime }{n}}^{-}\left(x,y\right),\psi_{\left({\;}^{\prime \prime }E\times F\right)\overset{\prime }{n}}^{+}\left(x,y\right)\right]e^{\left[\xi_{\left({\;}^{\prime \prime }E\times F\right)\overset{\prime }{n}}^{-}\left(x,y\right),\xi_{\left({\;}^{\prime \prime }E\times F\right)\overset{\prime }{n}}^{+}\left(x,y\right)\right]2\pi l} \end{array}\right)x\in^{\prime \prime }\text{Eandy}\in F\right\}, \end{array}$$

where

(14) $$\begin{array}{c} \psi_{\left(E\times F\right)m}^{-}\left(x,y\right)=\min\left\{\psi_{\left(E\right)m}^{-}\left(x\right),\psi_{\left(F\right)m}^{-}\left(y\right)\right\},\psi_{\left(E\times F\right)m}^{+}\left(x,y\right)=\min\left\{\psi_{\left(E\right)m}^{+}\left(x\right),\psi_{\left(F\right)m}^{+}\left(y\right)\right\},\\ \xi_{\left(E\times F\right)m}^{-}\left(x,y\right)=\min\left\{\xi_{\left(E\right)m}^{-}\left(x\right),\xi_{\left(F\right)m}^{-}\left(y\right)\right\}\text{ and }\;\xi_{\left(E\times F\right)m}^{+}\left(x,y\right)=\min\left\{\xi_{\left(E\right)m}^{+}\left(x\right),\xi_{\left(F\right)m}^{+}\left(y\right)\right\},\\ \psi_{\left(E\times F\right)a}^{-}\left(x,y\right)=\min\left\{\psi_{\left(E\right)a}^{-}\left(x\right),\psi_{\left(F\right)a}^{-}\left(x\right)\right\}\text{ and }\psi_{\left(E\times F\right)a}^{+}\left(x,y\right)=\min\left\{\psi_{\left(E\right)a}^{+}\left(x\right),\psi_{\left(F\right)a}^{+}\left(y\right)\right\},\\ \xi_{\left(E\times F\right)a}^{-}\left(x,y\right)=\min\left\{\xi_{\left(E\right)a}^{-}\left(x\right),\xi_{\left(F\right)a}^{-}\left(y\right)\right\}\text{ and }\xi_{\left(E\times F\right)a}^{+}\left(x,y\right)=\min\left\{\xi_{\left(E\right)a}^{+}\left(x\right),\xi_{\left(F\right)a}^{+}\left(y\right)\right\},\\ \psi_{\left(E\times F\right)n}^{-}\left(x,y\right)=\min\left\{\psi_{\left(E\right)n}^{-}\left(x\right),\psi_{\left(F\right)n}^{-}\left(x\right)\right\},\psi_{\left(E\times F\right)n}^{+}\left(x,y\right)=\min\left\{\psi_{\left(E\right)n}^{+}\left(x\right),\psi_{\left(F\right)n}^{+}\left(y\right)\right\},\\ \xi_{\left(E\times F\right)n}^{-}\left(x,y\right)=\min\left\{\xi_{\left(E\right)n}^{-}\left(x\right),\xi_{\left(F\right)n}^{-}\left(y\right)\right\}\text{ and }\xi_{\left(E\times F\right)n}^{+}\left(x,y\right)=\min\left\{\xi_{\left(E\right)n}^{+}\left(x\right),\xi_{\left(F\right)n}^{+}\left(y\right)\right\}. \end{array}$$

**Example 1 .**

Suppose that *E* is an IVCT-spherical-FS on *H* defined as follows for *n* = 8:

(15) $$\begin{array}{c} E=\left\{\begin{array}{c} \left(x,\left[0.51,0.61\right]e^{\left[0.60,0.71\right]2\pi l},\left[0.41,0.52\right]e^{\left[0.47,0.49\right]2\pi l},{\left[0.46,0.49\right]}^{\left[0.37,0.45\right]2\pi l}\right),\\ \left(y,\left[0.54,0.57\right]e^{\left[0.61,0.72\right]2\pi l},\left[0.42,0.54\right]e^{\left[0.48,0.51\right]2\pi l},\left[0.61,0.71\right]e^{\left[0.64,0.72\right]2\pi l}\right)\\ \left(z,\left[0.81,0.82\right]e^{\left[0.62,0.71\right]2\pi l},\left[0.71,0.84\right]e^{\left[0.63,0.73\right]2\pi l},\left[0.84,0.91\right]e^{\left[0.64,0.69\right]2\pi l}\right), \end{array},\right\}. \end{array}$$

Then the Cartesian product of *E* × *E* is defined as

(16) $$\begin{array}{c} E\times E=\left\{\begin{array}{c} \left(\left(x,x\right),\left[0.51,0.61\right]e^{\left[0.61,0.71\right]2\pi l},\left[0.41,0.52\right]e^{\left[0.47,0.49\right]2\pi l},\left[0.46,0.49\right]e^{\left[0.37,0.45\right]2\pi l}\right),\\ \left(\left(x,y\right),\left[0.51,0.57\right]e^{\left[0.60,0.71\right]2\pi l},\left[0.41,0.52\right]e^{\left[0.47,0.49\right]2\pi l},\left[0.61,0.71\right]e^{\left[0.64,0.72\right]2\pi l}\right),\\ \left(\left(x,z\right),\left[0.51,0.61\right]e^{\left[0.61,0.71\right]2\pi l},\left[0.41,0.52\right]e^{\left[0.47,0.49\right]2\pi l},\left[0.84,0.91\right]e^{\left[0.64,0.69\right]2\pi l}\right),\\ \left(\left(y,x\right),\left[0.51,0.57\right]e^{\left[0.60,0.71\right]2\pi l},\left[0.41,0.52\right]e^{\left[0.47,0.49\right]2\pi l},\left[0.61,0.71\right]e^{\left[0.64,0.72\right]2\pi l}\right),\\ \left(\left(y,y\right),\left[0.54,0.57\right]e^{\left[0.61,0.72\right]2\pi l},\left[0.42,0.54\right]e^{\left[0.48,0.51\right]2\pi l},\left[0.61,0.71\right]e^{\left[0.64,0.72\right]2\pi l}\right),\\ \left(\left(y,z\right),\left[0.54,0.57\right]e^{\left[0.61,0.71\right]2\pi l},\left[0.54,0.57\right]e^{\left[0.61,0.72\right]2\pi l},\left[0.84,0.91\right]e^{\left[0.64,0.72\right]2\pi l}\right),\\ \left(\left(z,x\right),\left[0.51,0.61\right]e^{\left[0.61,0.71\right]2\pi l},\left[0.41,0.52\right]e^{\left[0.47,0.49\right]2\pi l},\left[0.84,0.91\right]e^{\left[0.64,0.69\right]2\pi l}\right),\\ \left(\left(z,y\right),\left[0.54,0.57\right]e^{\left[0.61,0.72\right]2\pi l},\left[0.42,0.54\right]e^{\left[0.48,0.51\right]2\pi l},\left[0.84,0.91\right]e^{\left[0.64,0.72\right]2\pi l}\right),\\ \left(\left(z,z\right),\left[0.81,0.82\right]e^{\left[0.62,0.71\right]2\pi l},\left[0.71,0.84\right]e^{\left[0.63,0.73\right]2\pi l},\left[0.84,0.91\right]e^{\left[0.64,0.69\right]2\pi l}\right) \end{array}\right\}. \end{array}$$

**Definition 10 .**

An interval-valued complex T-spherical fuzzy relation *R* (IVCT-spherical-FR) is a subset of the any Cartesian product of two IVCT-spherical-FSs.

**Example 2 .**

Suppose that *E* and *F* are two IVCT-spherical-FSs for *n*=8.

(17) $$\begin{array}{c} E=\left\{\begin{array}{c} \left(x,\left[0.51,0.61\right]e^{\left[0.60,0.71\right]2\pi \iota },\left[0.41,0.52\right]e^{\left[0.47,0.49\right]2\pi \iota },\left[0.46,0.49\right]e^{\left[0.37,0.45\right]2\pi \iota }\right),\\ \left(y,\left[0.54,0.57\right]e^{\left[0.61,0.72\right]2\pi \iota },\left[0.42,0.54\right]e^{\left[0.48,0.51\right]2\pi \iota },\left[0.61,0.71\right]e^{\left[0.64,0.72\right]2\pi \iota }\right),\\ \left(z,\left[0.81,0.82\right]e^{\left[0.62,0.71\right]2\pi \iota },\left[0.71,0.84\right]e^{\left[0.63,0.73\right]2\pi \iota },\left[0.84,0.91\right]e^{\left[0.64,0.69\right]2\pi \iota }\right) \end{array}\right\}\text{and},\\ F=\left\{\begin{array}{c} \left(r,\left[0.46,0.53\right]e^{\left[0.59,0.56\right]2\pi l},\left[0.51,0.56\right]e^{\left[0.43,0.47\right]2\pi l},\left[0.36,0.43\right]e^{\left[0.39,0.49\right]2\pi l}\right),\\ \left(s,\left[0.52,0.59\right]e^{\left[0.63,0.71\right]2\pi l},\left[0.44,0.53\right]e^{\left[0.45,0.50\right]2\pi l},\left[0.59,0.67\right]e^{\left[0.61,0.69\right]2\pi l}\right),\\ \left(t,\left[0.79,0.81\right]e^{\left[0.61,0.73\right]2\pi l},\left[0.69,0.83\right]e^{\left[0.64,0.74\right]2\pi l},\left[0.83,0.90\right]e^{\left[0.63,0.67\right]2\pi l}\right) \end{array}\right\}. \end{array}$$

Then the Cartesian product of *E* × *F* is given as

(18) $$\begin{array}{c} E\times F=\left\{\begin{array}{c} \left(\left(x,r\right),\left[0.46,0.53\right]e^{\left[0.59,0.61\right]2\pi l},\left[0.41,0.52\right]e^{\left[0.43,0.47\right]2\pi l},\left[0.46,0.49\right]e^{\left[0.39,0.49\right]2\pi l}\right),\\ \left(\left(x,s\right),\left[0.51,0.59\right]e^{\left[0.60,0.71\right]2\pi l},\left[0.41,0.52\right]e^{\left[0.45,0.49\right]2\pi l},\left[0.59,0.67\right]e^{\left[0.61,0.69\right]2\pi l}\right),\\ \left(\left(x,t\right),\left[0.51,0.61\right]e^{\left[0.60,0.71\right]2\pi l},\left[0.41,0.52\right]e^{\left[0.47,0.49\right]2\pi l},\left[0.83,0.90\right]e^{\left[0.63,0.67\right]2\pi l}\right),\\ \left(\left(y,r\right),\left[0.46,0.53\right]e^{\left[0.59,0.61\right]2\pi l},\left[0.42,0.54\right]e^{\left[0.43,0.47\right]2\pi l},\left[0.61,0.71\right]e^{\left[0.64,0.72\right]2\pi l}\right),\\ \left(\left(y,s\right),\left[0.52,0.57\right]e^{\left[0.61,0.71\right]2\pi l},\left[0.42,0.53\right]e^{\left[0.45,0.50\right]2\pi l},\left[0.61,0.71\right]e^{\left[0.64,0.72\right]2\pi l}\right),\\ \left(\left(y,t\right),\left[0.54,0.57\right]e^{\left[0.61,0.72\right]2\pi l},\left[0.42,0.54\right]e^{\left[0.63,0.73\right]2\pi l},\left[0.83,0.90\right]e^{\left[0.64,0.69\right]2\pi l}\right),\\ \left(\left(Z,r\right),\left[0.46,0.53\right]e^{\left[0.59,0.61\right]2\pi l},\left[0.51,0.56\right]e^{\left[0.43,0.47\right]2\pi l},\left[0.84,0.91\right]e^{\left[0.64,0.69\right]2\pi l}\right),\\ \left(\left(Z,s\right),\left[0.52,0.59\right]e^{\left[0.62,0.71\right]2\pi l},\left[0.44,0.53\right]e^{\left[0.45,0.50\right]2\pi l},\left[0.84,0.91\right]e^{\left[0.64,0.69\right]2\pi l}\right),\\ \left(\left(Z,t\right),\left[0.79,0.81\right]e^{\left[0.61,0.71\right]2\pi l},\left[0.69,0.83\right]e^{\left[0.63,0.73\right]2\pi l},\left[0.84,0.91\right]e^{\left[0.64,0.69\right]2\pi l}\right), \end{array}\right\}. \end{array}$$

Then the subset *R* ⊂ *E* × *F* is called IVCT-spherical-FR given as

(19) $$\begin{array}{c} R=\left\{\begin{array}{c} \left(\left(x,s\right),\left[0.51,0.59\right]e^{\left[0.60,0.71\right]2\pi l},\left[0.41,0.52\right]e^{\left[0.45,0.49\right]2\pi l},\left[0.59,0.67\right]e^{\left[0.61,0.69\right]2\pi l}\right),\\ \left(\left(y,r\right),\left[0.46,0.53\right]e^{\left[0.59,0.61\right]2\pi l},\left[0.42,0.54\right]e^{\left[0.43,0.47\right]2\pi l},\left[0.61,0.71\right]e^{\left[0.64,0.72\right]2\pi l}\right),\\ \left(\left(y,t\right),\left[0.54,0.57\right]e^{\left[0.61,0.72\right]2\pi l},\left[0.42,0.54\right]e^{\left[0.63,0.73\right]2\pi l},\left[0.83,0.90\right]e^{\left[0.64,0.69\right]2\pi l}\right),\\ \left(\left(Z,r\right),\left[0.46,0.53\right]e^{\left[0.59,0.61\right]2\pi l},\left[0.51,0.56\right]e^{\left[0.43,0.47\right]2\pi l},\left[0.84,0.91\right]e^{\left[0.64,0.69\right]2\pi l}\right),\\ \left(\left(Z,t\right),\left[0.79,0.81\right]e^{\left[0.61,0.71\right]2\pi l},\left[0.69,0.83\right]e^{\left[0.63,0.73\right]2\pi l},\left[0.84,0.91\right]e^{\left[0.64,0.69\right]2\pi l}\right), \end{array}\right\}. \end{array}$$

**Figure 1:**
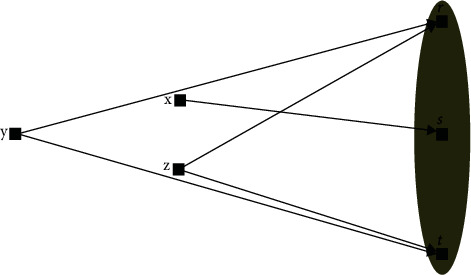
Interval-valued complex T-spherical fuzzy relation.

Figure 1 shows the relationship between the IVCT-spherical-FSs.

Table 1 shows the IVCT-spherical-FRs values discussed in the above diagram.

**Definition 11 .**

Let *E* be an IVCT-spherical-FS on *H* and let

(20) $$\begin{array}{c} \text{R}=\left\{\left(\begin{array}{c} \left(x,y\right)\left[\psi_{\left(\text{R}\right)\overset{\prime }{m}}^{-}\left(x,y\right),\psi_{\left(\text{R}\right)\overset{\prime }{m}}^{+}\left(x,y\right)\right]e^{\left[\xi_{\left(\text{R}\right)\overset{\prime }{m}}^{-}\left(x,y\right),\xi_{\left(\text{R}\right)\overset{\prime }{m}}^{+}\left(x,y\right)\right]2\pi l,}\\ \left(x,y\right)\left[\psi_{\left(\text{R}\right)\overset{\prime \prime }{m}}^{-}\left(x,y\right),\psi_{\left(\text{R}\right)\overset{\prime \prime }{m}}^{+}\left(x,y\right)\right]e^{\left[\xi_{\left(\text{R}\right)\overset{\prime \prime }{m}}^{-}\left(x,y\right),\xi_{\left(\text{R}\right)\overset{\prime \prime }{m}}^{+}\left(x,y\right)\right]2\pi l,}\\ \left(x,y\right),\left[\psi_{\left(\text{R}\right)\overset{\prime }{n}}^{-}\left(x,y\right),\psi_{\left(\text{R}\right)\overset{\prime }{n}}^{+}\left(x,y\right)\right]e^{\left[\xi_{\left(\text{R}\right)\overset{\prime }{n}}^{-}\left(x,y\right),\xi_{\left(\text{R}\right)\overset{\prime }{n}}^{+}\left(x,y\right)\right]2\pi l} \end{array}\right)x,y\in \text{R}\right\}\text{be an.} \end{array}$$

IVCT-spherical-FR on *E*. Then the inverse of IVCT-spherical-FR is denoted by R^−1^ and defined as

(21) $$\begin{array}{c} {\text{R}}^{-1}=\left\{\left(\begin{array}{c} \left(y,x\right)\left[\psi_{\left(\text{R}\right)\overset{\prime }{m}}^{-}\left(y,x\right),\psi_{\left(\text{R}\right)\overset{\prime }{m}}^{+}\left(y,x\right)\right]e^{\left[\xi_{\left(\text{R}\right)\overset{\prime }{m}}^{-}\left(y,x\right),\xi_{\left(\text{R}\right)\overset{\prime }{m}}^{+}\left(y,x\right)\right]2\pi l,}\\ \left(y,x\right)\left[\psi_{\left(\text{R}\right)\overset{\prime \prime }{\alpha }}^{-}\left(y,x\right),\psi_{\left(\text{R}\right)\overset{\prime \prime }{\alpha }}^{+}\left(y,x\right)\right]e^{\left[\xi_{\left(\text{R}\right)\overset{\prime \prime }{\alpha }}^{-}\left(y,x\right),\xi_{\left(\text{R}\right)\overset{\prime \prime }{\alpha }}^{+}\left(y,x\right)\right]2\pi l,}\\ \left(y,x\right),\left[\psi_{\left(\text{R}\right)\overset{\prime }{n}}^{-}\left(y,x\right),\psi_{\left(\text{R}\right)\overset{\prime }{n}}^{+}\left(y,x\right)\right]e^{\left[\xi_{\left(\text{R}\right)\overset{\prime }{n}}^{-}\left(y,x\right),\xi_{\left(\text{R}\right)\overset{\prime }{n}}^{+}\left(y,x\right)\right]2\pi l} \end{array}\right)y,x\in {\text{R}}^{-1}\right\}. \end{array}$$

**Example 3 .**

Suppose that

(22) $$\begin{array}{c} \text{R}=\left\{\begin{array}{c} \left(\left(x,y\right),\left[0.51,0.57\right]e^{\left[0.60,0.71\right]2\pi l},\left[0.41,0.52\right]e^{\left[0.47,0.49\right]2\pi l},\left[0.61,0.71\right]e^{\left[0.64,0.72\right]2\pi l}\right),\\ \left(\left(x,z\right),\left[0.51,0.61\right]e^{\left[0.60,0.71\right]2\pi l},\left[0.41,0.52\right]e^{\left[0.47,0.49\right]2\pi l},\left[0.84,0.91\right]e^{\left[0.64,0.72\right]2\pi l}\right),\\ \left(\left(y,z\right),\left[0.54,0.57\right]e^{\left[0.60,0.71\right]2\pi l},\left[0.42,0.54\right]e^{\left[0.48,0.51\right]2\pi l},\left[0.84,0.91\right]e^{\left[0.64,0.72\right]2\pi l}\right), \end{array}\right\}, \end{array}$$

and let it be an IVCT-spherical-FR on an IVCT-spherical-FS on *E* for *n*=8.

(23) $$\begin{array}{c} E=\left\{\begin{array}{c} \left(\left(x\right),\left[0.51,0.61\right]e^{\left[0.60,0.71\right]2\pi l},\left[0.41,0.52\right]e^{\left[0.47,0.49\right]2\pi l},\left[0.46,0.49\right]e^{\left[0.37,0.45\right]2\pi l}\right),\\ \left(\left(y\right),\left[0.54,0.57\right]e^{\left[0.61,0.72\right]2\pi l},\left[0.42,0.54\right]e^{\left[0.48,0.51\right]2\pi l},\left[0.61,0.71\right]e^{\left[0.64,0.72\right]2\pi l}\right),\\ \left(\left(z\right),\left[0.81,0.82\right]e^{\left[0.62,0.71\right]2\pi l},\left[0.71,0.84\right]e^{\left[0.63,0.73\right]2\pi l},\left[0.84,0.91\right]e^{\left[0.64,0.69\right]2\pi l}\right), \end{array}\right\}\text{ then}. \end{array}$$

the inverse relation of R is defined as

(24) $$\begin{array}{c} {\text{R}}^{-1}=\left\{\begin{array}{c} \left(\left(y,x\right),\left[0.51,0.57\right]e^{\left[0.60,0.71\right]2\pi l},\left[0.41,0.52\right]e^{\left[0.47,0.49\right]2\pi l},\left[0.61,0.71\right]e^{\left[0.64,0.72\right]2\pi l}\right),\\ \left(\left(z,x\right),\left[0.51,0.61\right]e^{\left[0.60,0.71\right]2\pi l},\left[0.41,0.52\right]e^{\left[0.47,0.49\right]2\pi l},\left[0.84,0.91\right]e^{\left[0.64,0.69\right]2\pi l}\right),\\ \left(\left(z,y\right),\left[0.54,0.57\right]e^{\left[0.60,0.71\right]2\pi l},\left[0.42,0.54\right]e^{\left[0.48,0.51\right]2\pi l},\left[0.84,0.91\right]e^{\left[0.64,0.72\right]2\pi l}\right), \end{array}\right\}. \end{array}$$

**Definition 12 .**

The IVCT-spherical-FR R_1_ is known as IVCT-spherical-F reflexive relation if

(25) $$\begin{array}{c} \left\{\left(\begin{array}{c} x,\left[\psi_{\left({\;}^{\prime \prime }E\right)\overset{\prime }{m}}^{-}\left(x\right),\psi_{\left({\;}^{\prime \prime }E\right)\overset{\prime }{m}}^{+}\left(x\right)\right]e^{\left[\xi_{\left({\;}^{\prime \prime }E\right)\overset{\prime }{m}}^{-}\left(x\right),\xi_{\left({\;}^{\prime \prime }E\right)\overset{\prime }{m}}^{+}\left(x\right)\right]2\pi l}\\ x,\left[\psi_{\left({\;}^{\prime \prime }E\right)\overset{\prime }{\alpha }}^{-}\left(x\right),\psi_{\left({\;}^{\prime \prime }E\right)\overset{\prime }{\alpha }}^{+}\left(x\right)\right]e^{\left[\xi_{\left({\;}^{\prime \prime }E\right)\overset{\prime }{\alpha }}^{-}\left(x\right),\xi_{\left({\;}^{\prime \prime }E\right)\overset{\prime }{\alpha }}^{+}\left(x\right)\right]2\pi l}\\ x,\left[\psi_{\left({\;}^{\prime \prime }E\right)\overset{\prime }{n}}^{-}\left(x\right),\psi_{\left({\;}^{\prime \prime }E\right)\overset{\prime }{n}}^{+}\left(x\right)\right]e^{\left[\xi_{\left({\;}^{\prime \prime }E\right)\overset{\prime }{n}}^{-}\left(x\right),\xi_{\left({\;}^{\prime \prime }E\right)\overset{\prime }{n}}^{+}\left(x\right)\right]2\pi l} \end{array}\right)\in E\right\}\\ ⟹\left\{\left(\begin{array}{c} \left(x,x\right)\left[\psi_{R\left({\;}^{\prime \prime }E\right)\overset{\prime }{m}}^{-}\left(x,x\right),\psi_{R\left({\;}^{\prime \prime }E\right)\overset{\prime }{m}}^{+}\left(x,x\right)\right]e^{\left[\xi_{R\left({\;}^{\prime \prime }E\right)\overset{\prime }{m}}^{-}\left(x,x\right),\xi_{R\left({\;}^{\prime \prime }E\right)\overset{\prime }{m}}^{+}\left(x,x\right)\right]2\pi l}\\ \left[\psi_{R\left({\;}^{\prime \prime }E\right)\overset{\prime }{\alpha }}^{-}\left(x,x\right),\psi_{RR\left({\;}^{\prime \prime }E\right)\overset{\prime }{\alpha }}^{+}\left(x,x\right)\right]e^{\left[\xi_{R\left({\;}^{\prime \prime }E\right)\overset{\prime }{\alpha }}^{-}\left(x,x\right),\xi_{\left({\;}^{\prime \prime }E\right)\overset{\prime }{\alpha }}^{+}\left(x,x\right)\right]2\pi l}\\ \left[\psi_{R\left({\;}^{\prime \prime }E\right)\overset{\prime }{n}}^{-}\left(x,x\right),\psi_{R\left({\;}^{\prime \prime }E\right)\overset{\prime }{n}}^{+}\left(x,x\right)\right]e^{\left[\xi_{R\left({\;}^{\prime \prime }E\right)\overset{\prime }{n}}^{-}\left(x,x\right),\xi_{R\left({\;}^{\prime \prime }E\right)\overset{\prime }{n}}^{+}\left(x,x\right)\right]2\pi l} \end{array}\right)\right.\in R_{1}. \end{array}$$

On the other hand, R_2_ is known as an IVCT-spherical-F irreflexive relation if

(26) $$\begin{array}{c} \left\{\left(\begin{array}{c} x,\left[\psi_{\left({\;}^{\prime \prime }E\right)\overset{\prime }{m}}^{-}\left(x\right),\psi_{\left({\;}^{\prime \prime }E\right)\overset{\prime }{m}}^{+}\left(x\right)\right]e^{\left[\xi_{\left({\;}^{\prime \prime }E\right)\overset{\prime }{m}}^{-}\left(x\right),\xi_{\left({\;}^{\prime \prime }E\right)\overset{\prime }{m}}^{+}\left(x\right)\right]2\pi l}\\ x,\left[\psi_{\left({\;}^{\prime \prime }E\right)\overset{\prime }{\alpha }}^{-}\left(x\right),\psi_{\left({\;}^{\prime \prime }E\right)\overset{\prime }{\alpha }}^{+}\left(x\right)\right]e^{\left[\xi_{\left({\;}^{\prime \prime }E\right)\overset{\prime }{\alpha }}^{-}\left(x\right),\xi_{\left({\;}^{\prime \prime }E\right)\overset{\prime }{\alpha }}^{+}\left(x\right)\right]2\pi l}\\ x,\left[\psi_{\left({\;}^{\prime \prime }E\right)\overset{\prime }{n}}^{-}\left(x\right),\psi_{\left({\;}^{\prime \prime }E\right)\overset{\prime }{n}}^{+}\left(x\right)\right]e^{\left[\xi_{\left({\;}^{\prime \prime }E\right)\overset{\prime }{n}}^{-}\left(x\right),\xi_{\left({\;}^{\prime \prime }E\right)\overset{\prime }{n}}^{+}\left(x\right)\right]2\pi l} \end{array}\right)\right.\in E\\ ⟹\left\{\left(\begin{array}{c} \left(x,x\right)\left[\psi_{R\left({\;}^{\prime \prime }E\right)\overset{\prime }{m}}^{-}\left(x,x\right),\psi_{R\left({\;}^{\prime \prime }E\right)\overset{\prime }{m}}^{+}\left(x,x\right)\right]e^{\left[\xi_{R\left({\;}^{\prime \prime }E\right)\overset{\prime }{m}}^{-}\left(x,x\right),\xi_{R\left({\;}^{\prime \prime }E\right)\overset{\prime }{m}}^{+}\left(x,x\right)\right]2\pi l}\\ \left[\psi_{R\left({\;}^{\prime \prime }E\right)\overset{\prime }{\alpha }}^{-}\left(x,x\right),\psi_{R\left({\;}^{\prime \prime }E\right)\overset{\prime }{\alpha }}^{+}\left(x,x\right)\right]e^{\left[\xi_{R\left({\;}^{\prime \prime }E\right)\overset{\prime }{\alpha }}^{-}\left(x,x\right),\xi_{R\left({\;}^{\prime \prime }E\right)\overset{\prime }{\alpha }}^{+}\left(x,x\right)\right]2\pi l}\\ \left[\psi_{R\left({\;}^{\prime \prime }E\right)\overset{\prime }{n}}^{-}\left(x,x\right),\psi_{R\left({\;}^{\prime \prime }E\right)\overset{\prime }{n}}^{+}\left(x,x\right)\right]e^{\left[\xi_{R\left({\;}^{\prime \prime }E\right)\overset{\prime }{n}}^{-}\left(x,x\right),\xi_{R\left({\;}^{\prime \prime }E\right)\overset{\prime }{n}}^{+}\left(x,x\right)\right]2\pi l} \end{array}\right)\right.\in R_{2}. \end{array}$$

**Example 4 .**

Let *E* be an IVCT-spherical-FS for *n*=8.

(27) $$\begin{array}{c} \text{“}E=\left\{\begin{array}{c} \left(x,\left[0.51,0.61\right]e^{\left[0.60,0.71\right]2\pi \iota },\left[0.41,0.52\right]e^{\left[0.47,0.49\right]2\pi \iota },\left[0.46,0.49\right]e^{\left[0.37,0.45\right]2\pi \iota }\right),\\ \left(y,\left[0.54,0.57\right]e^{\left[0.61,0.72\right]2\pi \iota },\left[0.42,0.54\right]e^{\left[0.48,0.51\right]2\pi \iota },\left[0.61,0.71\right]e^{\left[0.64,0.72\right]2\pi \iota }\right),\\ \left(z,\left[0.81,0.82\right]e^{\left[0.62,0.71\right]2\pi \iota },\left[0.71,0.84\right]e^{\left[0.63,0.73\right]2\pi \iota },\left[0.84,0.91\right]e^{\left[0.64,0.69\right]2\pi \iota }\right) \end{array}\right\}. \end{array}$$

Then, the Cartesian product is

(28) $$\begin{array}{c} \text{“}E\times E=\left\{\begin{array}{c} \left(\left(x,x\right),\left[0.51,0.61\right]e^{\left[0.60,0.71\right]2\pi \iota },\left[0.41,0.52\right]e^{\left[0.47,0.49\right]2\pi \iota },\left[0.46,0.49\right]e^{\left[0.37,0.45\right]2\pi \iota }\right),\\ \left(\left(x,y\right),\left[0.51,0.57\right]e^{\left[0.60,0.71\right]2\pi \iota },\left[0.41,0.52\right]e^{\left[0.47,0.49\right]2\pi \iota },\left[0.61,0.71\right]e^{\left[0.64,0.72\right]2\pi \iota }\right),\\ \left(\left(x,z\right),\left[0.51,0.61\right]e^{\left[0.60,0.71\right]2\pi \iota },\left[0.41,0.52\right]e^{\left[0.47,0.49\right]2\pi \iota },\left[0.84,0.91\right]e^{\left[0.64,0.69\right]2\pi \iota }\right),\\ \left(\left(y,x\right),\left[0.51,0.57\right]e^{\left[0.60,0.71\right]2\pi \iota },\left[0.41,0.52\right]e^{\left[0.47,0.49\right]2\pi \iota },\left[0.61,0.71\right]e^{\left[0.64,0.72\right]2\pi \iota }\right),\\ \left(\left(y,y\right),\left[0.54,0.57\right]e^{\left[0.61,0.72\right]2\pi \iota },\left[0.42,0.54\right]e^{\left[0.48,0.51\right]2\pi \iota },\left[0.61,0.71\right]e^{\left[0.64,0.72\right]2\pi \iota }\right),\\ \left(\left(y,z\right),\left[0.54,0.57\right]e^{\left[0.61,0.71\right]2\pi \iota },\left[0.42,0.54\right]e^{\left[0.48,0.51\right]2\pi \iota },\left[0.84,0.91\right]e^{\left[0.64,0.72\right]2\pi \iota }\right),\\ \left(\left(z,x\right),\left[0.51,0.61\right]e^{\left[0.60,0.71\right]2\pi \iota },\left[0.41,0.52\right]e^{\left[0.47,0.49\right]2\pi \iota },\left[0.84,0.91\right]e^{\left[0.64,0.69\right]2\pi \iota }\right),\\ \left(\left(z,y\right),\left[0.54,0.57\right]e^{\left[0.61,0.71\right]2\pi \iota },\left[0.42,0.54\right]e^{\left[0.48,0.51\right]2\pi \iota },\left[0.84,0.91\right]e^{\left[0.64,0.72\right]2\pi \iota }\right),\\ \left(\left(z,z\right),\left[0.81,0.82\right]e^{\left[0.62,0.71\right]2\pi \iota },\left[0.71,0.84\right]e^{\left[0.63,0.73\right]2\pi \iota },\left[0.84,0.91\right]e^{\left[0.64,0.69\right]2\pi \iota }\right) \end{array}\right\}. \end{array}$$

Then, the IVCT-spherical-F reflexive relation R_1_ is

(29) $$\begin{array}{c} {\text{R}}_{1\;}=\left\{\begin{array}{c} \left(\left(x,x\right),\left[0.51,0.61\right]e^{\left[0.60,0.71\right]2\pi \iota },\left[0.41,0.52\right]e^{\left[0.47,0.49\right]2\pi \iota },\left[0.46,0.49\right]e^{\left[0.37,0.45\right]2\pi \iota }\right),\\ \left(\left(x,z\right),\left[0.51,0.61\right]e^{\left[0.60,0.71\right]2\pi \iota },\left[0.41,0.52\right]e^{\left[0.47,0.49\right]2\pi \iota },\left[0.84,0.91\right]e^{\left[0.64,0.69\right]2\pi \iota }\right),\\ \left(\left(y,y\right),\left[0.54,0.57\right]e^{\left[0.61,0.72\right]2\pi \iota },\left[0.42,0.54\right]e^{\left[0.48,0.51\right]2\pi \iota },\left[0.61,0.71\right]e^{\left[0.64,0.72\right]2\pi \iota }\right),\\ \left(\left(z,y\right),\left[0.54,0.57\right]e^{\left[0.61,0.71\right]2\pi \iota },\left[0.42,0.54\right]e^{\left[0.48,0.51\right]2\pi \iota },\left[0.84,0.91\right]e^{\left[0.64,0.72\right]2\pi \iota }\right),\\ \left(\left(z,z\right),\left[0.81,0.82\right]e^{\left[0.62,0.71\right]2\pi \iota },\left[0.71,0.84\right]e^{\left[0.63,0.73\right]2\pi \iota },\left[0.84,0.91\right]e^{\left[0.64,0.69\right]2\pi \iota }\right) \end{array}\right\}. \end{array}$$

Also, the IVCT-spherical-F irreflexive relation R_2_ is

(30) $$\begin{array}{c} {\text{R}}_{2\;}=\left\{\begin{array}{c} \left(\left(x,y\right),\left[0.51,0.57\right]e^{\left[0.60,0.71\right]2\pi \iota },\left[0.41,0.52\right]e^{\left[0.47,0.49\right]2\pi \iota },\left[0.61,0.71\right]e^{\left[0.64,0.72\right]2\pi \iota }\right),\\ \left(\left(y,z\right),\left[0.54,0.57\right]e^{\left[0.61,0.71\right]2\pi \iota },\left[0.42,0.54\right]e^{\left[0.48,0.51\right]2\pi \iota },\left[0.84,0.91\right]e^{\left[0.64,0.72\right]2\pi \iota }\right),\\ \left(\left(z,y\right),\left[0.54,0.57\right]e^{\left[0.61,0.71\right]2\pi \iota },\left[0.42,0.54\right]e^{\left[0.48,0.51\right]2\pi \iota },\left[0.84,0.91\right]e^{\left[0.64,0.72\right]2\pi \iota }\right) \end{array}\right\}. \end{array}$$

**NOTE:** For convenience, throughout this article, *x* and (*x*, *y*) will be used to denote $\left(\begin{array}{c} x,\left[\psi_{\left(\text{“}E\right)\text{m}}^{-}\left(x\right),\psi_{\left(\text{“}E\right)\text{m}}^{+}\left(x\right)\right]e^{\left[\xi_{\left(\text{“}E\right)\text{m}}^{-}\left(x\right),\xi_{\left(\text{“}E\right)\text{m}}^{+}\left(x\right)\right]2\pi \iota },\\ \left[\psi_{\left(\text{“}E\right)a}^{-}\left(x\right),\psi_{\left(\text{“}E\right)a}^{+}\left(x\right)\right]e^{\left[\xi_{\left(\text{“}E\right)a}^{-}\left(x\right),\xi_{\left(\text{“}E\right)a}^{+}\left(x\right)\right]2\pi \iota },\left[\psi_{\left(\text{“}E\right)\text{n}\;}^{-}\left(x\right),\psi_{\left(\text{“}E\right)\text{n}\;}^{+}\left(x\right)\right]e^{\left[\xi_{\left(\text{“}E\right)\text{n}\;}^{-}\left(x\right),\xi_{\left(\text{“}E\right)\text{n}\;}^{+}\left(x\right)\right]2\pi \iota } \end{array}\right)$ and $\left(\begin{array}{c} \left(x,y\right),\left[\psi_{\left(\text{“}E\times \text{“}E\right)\text{m}}^{-}\left(x,y\right),\psi_{\left(\text{“}E\times \text{“}E\right)\text{m}}^{+}\left(x,y\right)\right]e^{\left[\xi_{\left(\text{“}E\times \text{“}E\right)\text{m}}^{-}\left(x,y\right),\xi_{\left(\text{“}E\times \text{“}E\right)\text{m}}^{+}\left(x,y\right)\right]2\pi \iota },\\ \left[\psi_{\left(\text{“}E\times \text{“}E\right)a}^{-}\left(x,y\right),\psi_{\left(\text{“}E\times \text{“}E\right)a}^{+}\left(x,y\right)\right]e^{\left[\xi_{\left(\text{“}E\times \text{“}E\right)a}^{-}\left(x,y\right),\xi_{\left(\text{“}E\times \text{“}E\right)a}^{+}\left(x,y\right)\right]2\pi \iota^{\prime }}\left[\psi_{\left(\text{“}E\times \text{“}E\right)\text{n}\;}^{-}\left(x,y\right),\psi_{\left(\text{“}E\times \text{“}E\right)\text{n}\;}^{+}\left(x,y\right)\right]e^{\left[\xi_{\left(\text{“}E\times \text{“}E\right)\text{n}\;}^{-}\left(x,y\right),\xi_{\left(\text{“}E\times \text{“}E\right)\text{n}\;}^{+}\left(x,y\right)\right]2\pi \iota } \end{array}\right)$, respectively; otherwise, it will be mentioned.

**Definition 13 .**

Consider that *F* is an IVCT-spherical-FS on the universal set *H* and let R_1_ be an IVCT-spherical-FR on *F*; then we have the following:

1. R_1_ is known as an IVCT-spherical symmetric-FR on *F* for all *x*, *y* ∈ *F*,  (*x*, *y*) ∈ R_1_⇒(*y*, *x*) ∈ R_1_
2. R_1_ is known as an IVCT-spherical antisymmetric-FR on *F* for all *x*, *y* ∈ *F*,  (*x*, *y*) ∈ *F*_1_ and (*y*, *x*) ∈ R_1_⇒*x*=*y*
3. R_1_ is known as an IVCT-spherical asymmetric-FR on *F* for all *x*, *y* ∈ *F*,  (*x*, *y*) ∈ R_1_⇒(*y*, *x*) ∉ R_1_
4. R_1_ is known as an IVCT-spherical complete-FR on *F* for all *x*, *y* ∈ *F*,  (*x*, *y*) ∈ R_1_ or (*y*, *x*) ∈ R_1_
5. R_1_ is known as an IVCT-spherical transitive-FR on *F* for all  *x*, *y*, *z* ∈ *F*,  (*x*, *y*) ∈ R_1_ and (*y*, *z*) ∈ R_1_⇒(*x*, *z*) ∈ R_1_
6. R_1_ is known as an IVCT-spherical equivalence-FR on *F* if  R_1_ is IVCT-spherical reflexive-FR, IVCT-spherical symmetric-FR, and IVCT-spherical transitive-FR on Ӻ
7. R_1_ is known as an IVCT-spherical preorder-FR on *F* if R_1_ is IVCT-spherical reflexive-FR and IVCT-spherical transitive-FR on Ӻ
8. R_1_ is known as an IVCT-spherical strict order-FR on *F* if R_1_ is IVCT-spherical irreflexive-FR and IVCT-spherical transitive-FR on Ӻ
9. R_1_ is known as an IVCT-spherical partial order-FR on *F* if R_1_ is IVCT-spherical preorder-FR and IVCT-spherical antisymmetric-FR on Ӻ
10. R_1_ is known as an IVCT-spherical linear order-FR on *F* if R_1_ is IVCT-spherical partial order-FR and IVCT-spherical complete-FR on Ӻ

**Example 5 .**

Let *F* be an IVCT-spherical fuzzy set for *n*=8.

(31) $$\begin{array}{c} Ӻ=\left\{\begin{array}{c} \left(x,\left[0.51,0.57\right]e^{\left[0.67,0.69\right]2\pi \iota },\left[0.46,0.49\right]e^{\left[0.61.0.67\right]2\pi \iota },\left[0.41,0.52\right]e^{\left[0.41,0.52\right]2\pi \iota }\right),\\ \left(y,\left[0.47,0.52\right]e^{\left[0.66,0.67\right]2\pi \iota },\left[0.49,0.53\right]e^{\left[0.62.0.65\right]2\pi \iota },\left[0.39,0.44\right]e^{\left[0.49,0.53\right]2\pi \iota }\right),\\ \left(z,\left[0.75,0.88\right]e^{\left[0.71,0.74\right]2\pi \iota },\left[0.79,0.89\right]e^{\left[0.64.0.68\right]2\pi \iota },\left[0.81,0.82\right]e^{\left[0.75,0.83\right]2\pi \iota }\right) \end{array}\right\}. \end{array}$$

Then, the Cartesian product is

(32) $$\begin{array}{c} Ӻ\times F=\left\{\begin{array}{c} \left(\left(x,x\right),\left[0.51,0.57\right]e^{\left[0.67,0.69\right]2\pi \iota },\left[0.46,0.49\right]e^{\left[0.61.0.67\right]2\pi \iota },\left[0.41,0.52\right]e^{\left[0.41,0.52\right]2\pi \iota }\right),\\ \left(\left(x,y\right),\left[0.47,0.52\right]e^{\left[0.66,0.67\right]2\pi \iota },\left[0.46,0.49\right]e^{\left[0.61.0.65\right]2\pi \iota },\left[0.41,0.52\right]e^{\left[0.49,0.52\right]2\pi \iota }\right),\\ \left(\left(x,z\right),\left[0.51,0.57\right]e^{\left[0.67,0.69\right]2\pi \iota },\left[0.46,0.49\right]e^{\left[0.61.0.67\right]2\pi \iota },\left[0.81,0.82\right]e^{\left[0.75,0.83\right]2\pi \iota }\right),\\ \left(\left(y,x\right),\left[0.47,0.52\right]e^{\left[0.66,0.67\right]2\pi \iota },\left[0.46,0.49\right]e^{\left[0.61.0.65\right]2\pi \iota },\left[0.41,0.52\right]e^{\left[0.49,0.52\right]2\pi \iota }\right),\\ \left(\left(y,y\right),\left[0.47,0.52\right]e^{\left[0.66,0.67\right]2\pi \iota },\left[0.49,0.53\right]e^{\left[0.62.0.65\right]2\pi \iota },\left[0.39,0.44\right]e^{\left[0.49,0.53\right]2\pi \iota }\right),\\ \left(\left(y,z\right),\left[0.47,0.52\right]e^{\left[0.66,0.67\right]2\pi \iota },\left[0.49,0.53\right]e^{\left[0.62.0.65\right]2\pi \iota },\left[0.81,0.82\right]e^{\left[0.75,0.83\right]2\pi \iota }\right),\\ \left(\left(z,x\right),\left[0.51,0.57\right]e^{\left[0.67,0.69\right]2\pi \iota },\left[0.46,0.49\right]e^{\left[0.61.0.67\right]2\pi \iota },\left[0.81,0.82\right]e^{\left[0.75,0.83\right]2\pi \iota }\right),\\ \left(\left(z,y\right),\left[0.47,0.52\right]e^{\left[0.66,0.67\right]2\pi \iota },\left[0.49,0.53\right]e^{\left[0.62.0.65\right]2\pi \iota },\left[0.81,0.82\right]e^{\left[0.75,0.83\right]2\pi \iota }\right),\\ \left(\left(z,z\right),\left[0.75,0.88\right]e^{\left[0.71,0.74\right]2\pi \iota },\left[0.79,0.89\right]e^{\left[0.64.0.68\right]2\pi \iota },\left[0.81,0.82\right]e^{\left[0.75,0.83\right]2\pi \iota }\right) \end{array}\right\}. \end{array}$$

(1) The IVCT-spherical symmetric-FR R_1_ on *F* is
  (33) $$\begin{array}{c} {\text{R}}_{1}=\left\{\begin{array}{c} \left(\left(x,y\right),\left[0.47,0.52\right]e^{\left[0.66,0.67\right]2\pi \iota },\left[0.46,0.49\right]e^{\left[0.61.0.65\right]2\pi \iota },\left[0.41,0.52\right]e^{\left[0.49,0.52\right]2\pi \iota }\right),\\ \left(\left(y,x\right),\left[0.47,0.52\right]e^{\left[0.66,0.67\right]2\pi \iota },\left[0.46,0.49\right]e^{\left[0.61.0.65\right]2\pi \iota },\left[0.41,0.52\right]e^{\left[0.49,0.52\right]2\pi \iota }\right),\\ \left(\left(y,z\right),\left[0.47,0.52\right]e^{\left[0.66,0.67\right]2\pi \iota },\left[0.49,0.53\right]e^{\left[0.62.0.65\right]2\pi \iota },\left[0.81,0.82\right]e^{\left[0.75,0.83\right]2\pi \iota }\right),\\ \left(\left(z,y\right),\left[0.47,0.52\right]e^{\left[0.66,0.67\right]2\pi \iota },\left[0.49,0.53\right]e^{\left[0.62.0.65\right]2\pi \iota },\left[0.81,0.82\right]e^{\left[0.75,0.83\right]2\pi \iota }\right) \end{array}\right\}. \end{array}$$
(2) The IVCT-spherical antisymmetric-FR R_2_ on *F* is
  (34) $$\begin{array}{c} {\text{R}}_{2}=\left\{\begin{array}{c} \left(\left(x,x\right),\left[0.51,0.57\right]e^{\left[0.67,0.69\right]2\pi \iota },\left[0.46,0.49\right]e^{\left[0.61.0.67\right]2\pi \iota },\left[0.41,0.52\right]e^{\left[0.41,0.52\right]2\pi \iota }\right),\\ \left(\left(y,y\right),\left[0.47,0.52\right]e^{\left[0.66,0.67\right]2\pi \iota },\left[0.49,0.53\right]e^{\left[0.62.0.65\right]2\pi \iota },\left[0.39,0.44\right]e^{\left[0.49,0.53\right]2\pi \iota }\right),\\ \left(\left(z,z\right),\left[0.75,0.88\right]e^{\left[0.71,0.74\right]2\pi \iota },\left[0.79,0.89\right]e^{\left[0.64.0.68\right]2\pi \iota },\left[0.81,0.82\right]e^{\left[0.75,0.83\right]2\pi \iota }\right) \end{array}\right\}. \end{array}$$
(3) The IVCT-spherical asymmetric-FR R_3_ on *F* is
  (35) $$\begin{array}{c} {\text{R}}_{3}=\left\{\begin{array}{c} \left(\left(x,y\right),\left[0.47,0.52\right]e^{\left[0.66,0.67\right]2\pi \iota },\left[0.46,0.49\right]e^{\left[0.61.0.65\right]2\pi \iota },\left[0.41,0.52\right]e^{\left[0.49,0.52\right]2\pi \iota }\right),\\ \left(\left(x,z\right),\left[0.51,0.57\right]e^{\left[0.67,0.69\right]2\pi \iota },\left[0.46,0.49\right]e^{\left[0.61.0.67\right]2\pi \iota },\left[0.81,0.82\right]e^{\left[0.75,0.83\right]2\pi \iota }\right),\\ \left(\left(y,z\right),\left[0.47,0.52\right]e^{\left[0.66,0.67\right]2\pi \iota },\left[0.49,0.53\right]e^{\left[0.62.0.65\right]2\pi \iota },\left[0.81,0.82\right]e^{\left[0.75,0.83\right]2\pi \iota }\right) \end{array}\right\}. \end{array}$$

**Theorem 1 .**

An IVCT-spherical-FR R is an IVCT-spherical symmetric FR on an IVCT-spherical-FS *F* if R=R^c^.

**Proof**

Suppose that  R=R^c^; then

(36) $$\begin{array}{c} \left(x,y\right)\in \text{R}\Rightarrow \left(y,x\right)\in {\text{R}}^{\text{c}}\Rightarrow \left(y,x\right)\in \text{R.} \end{array}$$

Therefore, R is an IVCT-spherical symmetric-FR on an IVCT-spherical-FS  *F*. Conversely, assume that R is an IVCT-spherical symmetric-FR on an IVCT-spherical-FS *F*; then

(37) $$\begin{array}{c} \left(x,y\right)\in \text{R}\Rightarrow \left(y,x\right)\in \text{R}. \end{array}$$

However, (*y*, *x*) ∈ R^c^⇒R=R^c^. Hence, the proof.

**Theorem 2 .**

If R_1_ and R_2_ are IVCT-spherical symmetric-FR; then R_1_∩R_2_ is an IVCT-spherical symmetric-FR.

**Proof**

Suppose that R_1_ and R_2_ are IVCT-spherical symmetric-FR on an IVCT-spherical-FS Ӻ. Then, by using the definition of IVCT-spherical-FR, R_1_ and R_2_ are subsets of Cartesian product *F* × *F*.R_1_⊆*F* × *F* and R_1_⊆*F* × *F*, so R_1_∩R_2_⊆*F* × *F*. Therefore, R_1_∩R_2_ is also an IVCT-spherical-FR on *F*.

(38) $$\begin{array}{c} \text{for all}\left(\begin{array}{c} \left(x,y\right)\left[\psi_{\left(\text{R}\right)\text{m}}^{-}\left(x,y\right),\psi_{\left(\text{R}\right)\text{m}}^{+}\left(x,y\right)\right]e^{\left[\xi_{\left(\text{R}\right)\text{m}}^{-}\left(x,y\right),\xi_{\left(\text{R}\right)\text{m}}^{+}\left(x,y\right)\right]2\pi \iota },\\ \left(x,y\right)\left[\psi_{\left(\text{R}\right)\text{a}}^{-}\left(x,y\right),\psi_{\left(\text{R}\right)\text{a}}^{+}\left(x,y\right)\right]e^{\left[\xi_{\left(\text{R}\right)\text{a}}^{-}\left(x,y\right),\xi_{\left(\text{R}\right)\text{a}}^{+}\left(x,y\right)\right]2\pi \iota },\\ \left(x,y\right)\left[\psi_{\left(\text{R}\right)\text{n}}^{-}\left(x,y\right),\psi_{\left(\text{R}\right)\text{n}}^{+}\left(x,y\right)\right]e^{\left[\xi_{\left(\text{R}\right)\text{n}}^{-}\left(x,y\right),\xi_{\left(\text{R}\right)\text{n}}^{+}\left(x,y\right)\right]2\pi \iota } \end{array}\right)\in {\text{R}}_{1}\cap {\text{R}}_{2}\\ \Rightarrow \left(\begin{array}{c} \left(x,y\right)\left[\psi_{\left(\text{R}\right)\text{m}}^{-}\left(x,y\right),\psi_{\left(\text{R}\right)\text{m}}^{+}\left(x,y\right)\right]e^{\left[\xi_{\left(\text{R}\right)\text{m}}^{-}\left(x,y\right),\xi_{\left(\text{R}\right)\text{m}}^{+}\left(x,y\right)\right]2\pi \iota },\\ \left(x,y\right)\left[\psi_{\left(\text{R}\right)\text{a}}^{-}\left(x,y\right),\psi_{\left(\text{R}\right)\text{a}}^{+}\left(x,y\right)\right]e^{\left[\xi_{\left(\text{R}\right)\text{a}}^{-}\left(x,y\right),\xi_{\left(\text{R}\right)\text{a}}^{+}\left(x,y\right)\right]2\pi \iota },\\ \left(x,y\right)\left[\psi_{\left(\text{R}\right)\text{n}}^{-}\left(x,y\right),\psi_{\left(\text{R}\right)\text{n}}^{+}\left(x,y\right)\right]e^{\left[\xi_{\left(\text{R}\right)\text{n}}^{-}\left(x,y\right),\xi_{\left(\text{R}\right)\text{n}}^{+}\left(x,y\right)\right]2\pi \iota } \end{array}\right)\in {\text{R}}_{1}\\ \left(\begin{array}{c} \left(x,y\right)\left[\psi_{\left(\text{R}\right)\text{m}}^{-}\left(x,y\right),\psi_{\left(\text{R}\right)\text{m}}^{+}\left(x,y\right)\right]e^{\left[\xi_{\left(\text{R}\right)\text{m}}^{-}\left(x,y\right),\xi_{\left(\text{R}\right)\text{m}}^{+}\left(x,y\right)\right]2\pi \iota },\\ \left(x,y\right)\left[\psi_{\left(\text{R}\right)\text{a}}^{-}\left(x,y\right),\psi_{\left(\text{R}\right)\text{a}}^{+}\left(x,y\right)\right]e^{\left[\xi_{\left(\text{R}\right)\text{a}}^{-}\left(x,y\right),\xi_{\left(\text{R}\right)\text{a}}^{+}\left(x,y\right)\right]2\pi \iota },\\ \left(x,y\right)\left[\psi_{\left(\text{R}\right)\text{n}}^{-}\left(x,y\right),\psi_{\left(\text{R}\right)\text{n}}^{+}\left(x,y\right)\right]e^{\left[\xi_{\left(\text{R}\right)\text{n}}^{-}\left(x,y\right),\xi_{\left(\text{R}\right)\text{n}}^{+}\left(x,y\right)\right]2\pi \iota } \end{array}\right)\in {\text{R}}_{2}. \end{array}$$

Also, R_1_and R_2_ are IVCT-spherical symmetric-FRs. Therefore,

(39) $$\begin{array}{c} \left(\begin{array}{c} \left(x,y\right)\left[\psi_{\left(\text{R}\right)\text{m}}^{-}\left(x,y\right),\psi_{\left(\text{R}\right)\text{m}}^{+}\left(x,y\right)\right]e^{\left[\xi_{\left(\text{R}\right)\text{m}}^{-}\left(x,y\right),\xi_{\left(\text{R}\right)\text{m}}^{+}\left(x,y\right)\right]2\pi \iota },\\ \left(x,y\right)\left[\psi_{\left(\text{R}\right)\text{a}}^{-}\left(x,y\right),\psi_{\left(\text{R}\right)\text{a}}^{+}\left(x,y\right)\right]e^{\left[\xi_{\left(\text{R}\right)\text{a}}^{-}\left(x,y\right),\xi_{\left(\text{R}\right)\text{a}}^{+}\left(x,y\right)\right]2\pi \iota },\\ \left(x,y\right)\left[\psi_{\left(\text{R}\right)\text{n}}^{-}\left(x,y\right),\psi_{\left(\text{R}\right)\text{n}}^{+}\left(x,y\right)\right]e^{\left[\xi_{\left(\text{R}\right)\text{n}}^{-}\left(x,y\right),\xi_{\left(\text{R}\right)\text{n}}^{+}\left(x,y\right)\right]2\pi \iota } \end{array}\right)\in {\text{R}}_{1}, \end{array}$$

and

(40) $$\begin{array}{c} \left(\begin{array}{c} \left(x,y\right)\left[\psi_{\left(\text{R}\right)\text{m}}^{-}\left(x,y\right),\psi_{\left(\text{R}\right)\text{m}}^{+}\left(x,y\right)\right]e^{\left[\xi_{\left(\text{R}\right)\text{m}}^{-}\left(x,y\right),\xi_{\left(\text{R}\right)\text{m}}^{+}\left(x,y\right)\right]2\pi \iota },\\ \left(x,y\right)\left[\psi_{\left(\text{R}\right)\text{a}}^{-}\left(x,y\right),\psi_{\left(\text{R}\right)\text{a}}^{+}\left(x,y\right)\right]e^{\left[\xi_{\left(\text{R}\right)\text{a}}^{-}\left(x,y\right),\xi_{\left(\text{R}\right)\text{a}}^{+}\left(x,y\right)\right]2\pi \iota },\\ \left(x,y\right)\left[\psi_{\left(\text{R}\right)\text{n}}^{-}\left(x,y\right),\psi_{\left(\text{R}\right)\text{n}}^{+}\left(x,y\right)\right]e^{\left[\xi_{\left(\text{R}\right)\text{n}}^{-}\left(x,y\right),\xi_{\left(\text{R}\right)\text{n}}^{+}\left(x,y\right)\right]2\pi \iota } \end{array}\right)\in {\text{R}}_{2}\\ \left(\begin{array}{c} \left(x,y\right)\left[\psi_{\left(\text{R}\right)\text{m}}^{-}\left(x,y\right),\psi_{\left(\text{R}\right)\text{m}}^{+}\left(x,y\right)\right]e^{\left[\xi_{\left(\text{R}\right)\text{m}}^{-}\left(x,y\right),\xi_{\left(\text{R}\right)\text{m}}^{+}\left(x,y\right)\right]2\pi \iota },\\ \left(x,y\right)\left[\psi_{\left(\text{R}\right)\text{a}}^{-}\left(x,y\right),\psi_{\left(\text{R}\right)\text{a}}^{+}\left(x,y\right)\right]e^{\left[\xi_{\left(\text{R}\right)\text{a}}^{-}\left(x,y\right),\xi_{\left(\text{R}\right)\text{a}}^{+}\left(x,y\right)\right]2\pi \iota },\\ \left(x,y\right)\left[\psi_{\left(\text{R}\right)\text{n}}^{-}\left(x,y\right),\psi_{\left(\text{R}\right)\text{n}}^{+}\left(x,y\right)\right]e^{\left[\xi_{\left(\text{R}\right)\text{n}}^{-}\left(x,y\right),\xi_{\left(\text{R}\right)\text{n}}^{+}\left(x,y\right)\right]2\pi \iota } \end{array}\right)\in {\text{R}}_{1}\cap {\text{R}}_{2}\\ \Rightarrow \left(\begin{array}{c} \left(x,y\right)\left[\psi_{\left(\text{R}\right)\text{m}}^{-}\left(x,y\right),\psi_{\left(\text{R}\right)\text{m}}^{+}\left(x,y\right)\right]e^{\left[\xi_{\left(\text{R}\right)\text{m}}^{-}\left(x,y\right),\xi_{\left(\text{R}\right)\text{m}}^{+}\left(x,y\right)\right]2\pi \iota },\\ \left(x,y\right)\left[\psi_{\left(\text{R}\right)\text{a}}^{-}\left(x,y\right),\psi_{\left(\text{R}\right)\text{a}}^{+}\left(x,y\right)\right]e^{\left[\xi_{\left(\text{R}\right)\text{a}}^{-}\left(x,y\right),\xi_{\left(\text{R}\right)\text{a}}^{+}\left(x,y\right)\right]2\pi \iota },\\ \left(x,y\right)\left[\psi_{\left(\text{R}\right)\text{n}}^{-}\left(x,y\right),\psi_{\left(\text{R}\right)\text{n}}^{+}\left(x,y\right)\right]e^{\left[\xi_{\left(\text{R}\right)\text{n}}^{-}\left(x,y\right),\xi_{\left(\text{R}\right)\text{n}}^{+}\left(x,y\right)\right]2\pi \iota } \end{array}\right)\in {\text{R}}_{1}\cap {\text{R}}_{2}. \end{array}$$

Hence, R_1_∩R_2_ is an IVCT-spherical symmetric- FR.

**Theorem 3 .**

If R_1_ is an IVCT-spherical equivalence-FR on an IVCT-spherical-FS *F*, then (*x*, *y*) ∈ R_1_, *iff* R_1_[u]=R_1_[*v*].

**Proof**

Suppose that (û, *v*) ∈ R_1_ and *w* ∈ R_1_[u], and R_1_(*w*, u) ∈ R_1_.

Now, by using the fact that an IVCT-spherical equivalence-FR is also an IVCT-spherical transitive-FR, (*w*, *v*) ∈ R_1_⇒*w* ∈ R_1_[*v*].

Thus,

(41) $$\begin{array}{c} {\text{R}}_{1}\left[\text{u}\right]\subseteq {\text{R}}_{1}\left[v\right]. \end{array}$$

As (*u*, *v*) ∈ R_1_, by using the fact that an IVCT-spherical equivalence-FR is also an IVCT-spherical symmetric-FR,

(42) $$\begin{array}{c} \left(\text{v},\text{u}\right)\in {\text{R}}_{1}. \end{array}$$

Additionally, assume that *w* ∈ R_1_[*v*]⇒(*w*, *v*) ∈ R_1_.

Now, again by using the fact that an IVCT-spherical equivalence-FR is also an IVCT-spherical transitive-FR,

(43) $$\begin{array}{c} \left(\text{w},\text{u}\right)\in {\text{R}}_{1}\Rightarrow \text{w}\in {\text{R}}_{1}\left[\text{u}\right]. \end{array}$$

Thus,

(44) $$\begin{array}{c} {\text{R}}_{1}\left[v\right]={\text{R}}_{1}\left[\text{u}\right]. \end{array}$$

Therefore, (41) and (44) give us R_1_[*v*]=R_1_[u].

Conversely, assume that R_1_[*v*]=R_1_[u],  *w* ∈ R_1_[u] and *w* ∈ R_1_[*v*]⇒(*w*, *v*) ∈ R_1_ and (*w*, *u*) ∈ R_1_.

Again, by using the fact that an IVCT-spherical equivalence-FR is also an IVCT-spherical symmetric-FR, (*w*, u) ∈ R_1_⇒(u, *w*) ∈ R_1_.

Now, by the definition of IVCT-spherical transitive-FR, (u, *w*) ∈ R_1_ and  (*w*, *v*) ∈ R_1_⇒(u, *v*) ∈ R_1_, which completes the proof.

**Example 6 .**

Let *F* be an IVCT-spherical fuzzy set for *n*=4.

(45) $$\begin{array}{c} Ӻ=\left\{\begin{array}{c} \left(x,\left[0.64,0.66\right]e^{\left[0.65,0.68\right]2\pi \iota },\left[0.58,0.64\right]e^{\left[0.61.0.67\right]2\pi \iota },\left[0.53,0.57\right]e^{\left[0.59,0.65\right]2\pi \iota }\right),\\ \left(y,\left[0.69,0.74\right]e^{\left[0.54,0.59\right]2\pi \iota },\left[0.64,0.67\right]e^{\left[0.69.0.72\right]2\pi \iota },\left[0.63,0.69\right]e^{\left[0.58,0.61\right]2\pi \iota }\right),\\ \left(z,\left[0.53,0.58\right]e^{\left[0.51,0.56\right]2\pi \iota },\left[0.52,0.57\right]e^{\left[0.61.0.65\right]2\pi \iota },\left[0.63,0.66\right]e^{\left[0.69,0.71\right]2\pi \iota }\right) \end{array}\right\}. \end{array}$$

Then, the Cartesian product is

(46) $$\begin{array}{c} Ӻ\times Ӻ=\left\{\begin{array}{c} \left(\left(x,x\right),\left[0.64,0.66\right]e^{\left[0.65,0.68\right]2\pi \iota },\left[0.58,0.64\right]e^{\left[0.61.0.67\right]2\pi \iota },\left[0.53,0.57\right]e^{\left[0.59,0.65\right]2\pi \iota }\right),\\ \left(\left(x,y\right),\left[0.64,0.66\right]e^{\left[0.54,0.59\right]2\pi \iota },\left[0.58,0.64\right]e^{\left[0.61.0.67\right]2\pi \iota },\left[0.63,0.69\right]e^{\left[0.58,0.61\right]2\pi \iota }\right),\\ \left(\left(x,z\right),\left[0.53,0.58\right]e^{\left[0.51,0.56\right]2\pi \iota },\left[0.52,0.57\right]e^{\left[0.61.0.65\right]2\pi \iota },\left[0.63,0.66\right]e^{\left[0.69,0.71\right]2\pi \iota }\right),\\ \left(\left(y,x\right),\left[0.64,0.66\right]e^{\left[0.54,0.59\right]2\pi \iota },\left[0.58,0.64\right]e^{\left[0.61.0.67\right]2\pi \iota },\left[0.63,0.69\right]e^{\left[0.58,0.61\right]2\pi \iota }\right),\\ \left(\left(y,y\right),\left[0.69,0.74\right]e^{\left[0.54,0.59\right]2\pi \iota },\left[0.64,0.67\right]e^{\left[0.69.0.72\right]2\pi \iota },\left[0.63,0.69\right]e^{\left[0.58,0.61\right]2\pi \iota }\right),\\ \left(\left(y,z\right),\left[0.53,0.58\right]e^{\left[0.51,0.56\right]2\pi \iota },\left[0.52,0.57\right]e^{\left[0.61.0.65\right]2\pi \iota },\left[0.63,0.69\right]e^{\left[0.69,0.71\right]2\pi \iota }\right),\\ \left(\left(z,x\right),\left[0.53,0.58\right]e^{\left[0.51,0.56\right]2\pi \iota },\left[0.52,0.57\right]e^{\left[0.61.0.65\right]2\pi \iota },\left[0.63,0.66\right]e^{\left[0.69,0.71\right]2\pi \iota }\right),\\ \left(\left(z,y\right),\left[0.53,0.58\right]e^{\left[0.51,0.56\right]2\pi \iota },\left[0.52,0.57\right]e^{\left[0.61.0.65\right]2\pi \iota },\left[0.63,0.69\right]e^{\left[0.69,0.71\right]2\pi \iota }\right),\\ \left(\left(z,z\right),\left[0.53,0.58\right]e^{\left[0.51,0.56\right]2\pi \iota },\left[0.52,0.57\right]e^{\left[0.61.0.65\right]2\pi \iota },\left[0.63,0.66\right]e^{\left[0.69,0.71\right]2\pi \iota }\right) \end{array}\right\}. \end{array}$$

(1) The IVCT-spherical transitive-FR R_1_ on *F* is
  (47) $$\begin{array}{c} {\text{R}}_{1}=\left\{\begin{array}{c} \left(\left(x,y\right),\left[0.64,0.66\right]e^{\left[0.54,0.59\right]2\pi \iota },\left[0.58,0.64\right]e^{\left[0.61.0.67\right]2\pi \iota },\left[0.63,0.69\right]e^{\left[0.58,0.61\right]2\pi \iota }\right),\\ \left(\left(y,z\right),\left[0.53,0.58\right]e^{\left[0.51,0.56\right]2\pi \iota },\left[0.52,0.57\right]e^{\left[0.61.0.65\right]2\pi \iota },\left[0.63,0.69\right]e^{\left[0.69,0.71\right]2\pi \iota }\right),\\ \left(\left(z,x\right),\left[0.53,0.58\right]e^{\left[0.51,0.56\right]2\pi \iota },\left[0.52,0.57\right]e^{\left[0.61.0.65\right]2\pi \iota },\left[0.63,0.66\right]e^{\left[0.69,0.71\right]2\pi \iota }\right) \end{array}\right\}. \end{array}$$
(2) The IVCT-spherical equivalence-FR R_2_ on *F* is
  (48) $$\begin{array}{c} {\text{R}}_{2}=\left\{\begin{array}{c} \left(\left(x,x\right),\left[0.64,0.66\right]e^{\left[0.65,0.68\right]2\pi \iota },\left[0.58,0.64\right]e^{\left[0.61.0.67\right]2\pi \iota },\left[0.53,0.57\right]e^{\left[0.59,0.65\right]2\pi \iota }\right),\\ \left(\left(y,z\right),\left[0.53,0.58\right]e^{\left[0.51,0.56\right]2\pi \iota },\left[0.52,0.57\right]e^{\left[0.61.0.65\right]2\pi \iota },\left[0.63,0.69\right]e^{\left[0.69,0.71\right]2\pi \iota }\right),\\ \left(\left(y,y\right),\left[0.69,0.74\right]e^{\left[0.54,0.59\right]2\pi \iota },\left[0.64,0.67\right]e^{\left[0.69.0.72\right]2\pi \iota },\left[0.63,0.69\right]e^{\left[0.58,0.61\right]2\pi \iota }\right),\\ \left(\left(z,y\right),\left[0.53,0.58\right]e^{\left[0.51,0.56\right]2\pi \iota },\left[0.52,0.57\right]e^{\left[0.61.0.65\right]2\pi \iota },\left[0.63,0.69\right]e^{\left[0.69,0.71\right]2\pi \iota }\right),\\ \left(\left(z,z\right),\left[0.53,0.58\right]e^{\left[0.51,0.56\right]2\pi \iota },\left[0.52,0.57\right]e^{\left[0.61.0.65\right]2\pi \iota },\left[0.63,0.66\right]e^{\left[0.69,0.71\right]2\pi \iota }\right) \end{array}\right\}. \end{array}$$

**Definition 14 .**

Suppose that R is an IVCT-spherical-FR; then the IVCT-spherical equivalence class of *x* is defined as R^*x*^={*y|*(*y*, *x*) ∈ R}.

**Example 7 .**

(49) $$\begin{array}{c} \text{R}=\left\{\begin{array}{c} \left(\left(x,x\right),\left[0.64,0.66\right]e^{\left[0.65,0.68\right]2\pi \iota },\left[0.58,0.64\right]e^{\left[0.61.0.67\right]2\pi \iota },\left[0.53,0.57\right]e^{\left[0.59,0.65\right]2\pi \iota }\right),\\ \left(\left(y,z\right),\left[0.53,0.58\right]e^{\left[0.51,0.56\right]2\pi \iota },\left[0.52,0.57\right]e^{\left[0.61.0.65\right]2\pi \iota },\left[0.63,0.69\right]e^{\left[0.69,0.71\right]2\pi \iota }\right),\\ \left(\left(y,y\right),\left[0.69,0.74\right]e^{\left[0.54,0.59\right]2\pi \iota },\left[0.64,0.67\right]e^{\left[0.69.0.72\right]2\pi \iota },\left[0.63,0.69\right]e^{\left[0.58,0.61\right]2\pi \iota }\right),\\ \left(\left(z,y\right),\left[0.53,0.58\right]e^{\left[0.51,0.56\right]2\pi \iota },\left[0.52,0.57\right]e^{\left[0.61.0.65\right]2\pi \iota },\left[0.63,0.69\right]e^{\left[0.69,0.71\right]2\pi \iota }\right),\\ \left(\left(z,z\right),\left[0.53,0.58\right]e^{\left[0.51,0.56\right]2\pi \iota },\left[0.52,0.57\right]e^{\left[0.61.0.65\right]2\pi \iota },\left[0.63,0.66\right]e^{\left[0.69,0.71\right]2\pi \iota }\right) \end{array}\right\}, \end{array}$$

and it is an IVCT-spherical-FR on an IVCT-spherical-FS for *n*=4.

(50) $$\begin{array}{c} Ӻ=\left\{\begin{array}{c} \left(x,\left[0.64,0.66\right]e^{\left[0.65,0.68\right]2\pi \iota },\left[0.58,0.64\right]e^{\left[0.61.0.67\right]2\pi \iota },\left[0.53,0.57\right]e^{\left[0.59,0.65\right]2\pi \iota }\right),\\ \left(y,\left[0.69,0.74\right]e^{\left[0.54,0.59\right]2\pi \iota },\left[0.64,0.67\right]e^{\left[0.69.0.72\right]2\pi \iota },\left[0.63,0.69\right]e^{\left[0.58,0.61\right]2\pi \iota }\right),\\ \left(z,\left[0.53,0.58\right]e^{\left[0.51,0.56\right]2\pi \iota },\left[0.52,0.57\right]e^{\left[0.61.0.65\right]2\pi \iota },\left[0.63,0.66\right]e^{\left[0.69,0.71\right]2\pi \iota }\right) \end{array}\right\}\text{,then} \end{array}$$

the IVCT-spherical fuzzy equivalence class of

(1) *x* modulo R is given as
  (51) $$\begin{array}{c} {\text{R}}^{x}=\left\{\left(x,\left[0.64,0.66\right]e^{\left[0.65,0.68\right]2\pi \iota },\left[0.58,0.64\right]e^{\left[0.61.0.67\right]2\pi \iota },\left[0.53,0.57\right]e^{\left[0.59,0.65\right]2\pi \iota }\right)\right\}. \end{array}$$
(2) *y* modulo R is given as
  (52) $$\begin{array}{c} {\text{R}}^{y}=\left\{\begin{array}{c} \left(z,\left[0.53,0.58\right]e^{\left[0.51,0.56\right]2\pi \iota },\left[0.52,0.57\right]e^{\left[0.61.0.65\right]2\pi \iota },\left[0.63,0.66\right]e^{\left[0.69,0.71\right]2\pi \iota }\right),\\ \left(y,\left[0.69,0.74\right]e^{\left[0.54,0.59\right]2\pi \iota },\left[0.64,0.67\right]e^{\left[0.69.0.72\right]2\pi \iota },\left[0.63,0.69\right]e^{\left[0.58,0.61\right]2\pi \iota }\right) \end{array}\right\}. \end{array}$$
(3) *z* modulo R is given as
  (53) $$\begin{array}{c} {\text{R}}^{z}=\left\{\begin{array}{c} \left(y,\left[0.69,0.74\right]e^{\left[0.54,0.59\right]2\pi \iota },\left[0.64,0.67\right]e^{\left[0.69.0.72\right]2\pi \iota },\left[0.63,0.69\right]e^{\left[0.58,0.61\right]2\pi \iota }\right),\\ \left(z,\left[0.53,0.58\right]e^{\left[0.51,0.56\right]2\pi \iota },\left[0.52,0.57\right]e^{\left[0.61.0.65\right]2\pi \iota },\left[0.63,0.66\right]e^{\left[0.69,0.71\right]2\pi \iota }\right) \end{array}\right\}. \end{array}$$

**Example 8 .**

Let *F* be an IVCT-spherical fuzzy set for *n*=5.

(54) $$\begin{array}{c} Ӻ=\left\{\begin{array}{c} \left(x,\left[0.64,0.70\right]e^{\left[0.65,0.71\right]2\pi \iota },\left[0.66,0.68\right]e^{\left[0.68.0.73\right]2\pi \iota },\left[0.69,0.72\right]e^{\left[0.61,0.74\right]2\pi \iota }\right),\\ \left(y,\left[0.62,0.67\right]e^{\left[0.68,0.72\right]2\pi \iota },\left[0.58,0.64\right]e^{\left[0.68.0.75\right]2\pi \iota },\left[0.62,0.69\right]e^{\left[0.71,0.78\right]2\pi \iota }\right),\\ \left(z,\left[0.58,0.73\right]e^{\left[0.61,0.78\right]2\pi \iota },\left[0.61,0.87\right]e^{\left[0.64.0.84\right]2\pi \iota },\left[0.59,0.65\right]e^{\left[0.61,0.75\right]2\pi \iota }\right) \end{array}\right\}. \end{array}$$

Then, the Cartesian product is

(55) $$\begin{array}{c} Ӻ\times Ӻ=\left\{\begin{array}{c} \left(\left(x,x\right),\left[0.64,0.70\right]e^{\left[0.65,0.71\right]2\pi \iota },\left[0.66,0.68\right]e^{\left[0.68.0.73\right]2\pi \iota },\left[0.69,0.72\right]e^{\left[0.61,0.74\right]2\pi \iota }\right),\\ \left(\left(x,y\right),\left[0.62,0.67\right]e^{\left[0.65,0.71\right]2\pi \iota },\left[0.58,0.64\right]e^{\left[0.68.0.73\right]2\pi \iota },\left[0.69,0.72\right]e^{\left[0.71,0.78\right]2\pi \iota }\right),\\ \left(\left(x,z\right),\left[0.58,0.70\right]e^{\left[0.61,0.71\right]2\pi \iota },\left[0.61,0.68\right]e^{\left[0.64.0.73\right]2\pi \iota },\left[0.69,0.72\right]e^{\left[0.61,0.75\right]2\pi \iota }\right),\\ \left(\left(y,x\right),\left[0.62,0.67\right]e^{\left[0.65,0.71\right]2\pi \iota },\left[0.58,0.64\right]e^{\left[0.68.0.73\right]2\pi \iota },\left[0.69,0.72\right]e^{\left[0.71,0.78\right]2\pi \iota }\right),\\ \left(\left(y,y\right),\left[0.62,0.67\right]e^{\left[0.68,0.72\right]2\pi \iota },\left[0.58,0.64\right]e^{\left[0.68.0.75\right]2\pi \iota },\left[0.62,0.69\right]e^{\left[0.71,0.78\right]2\pi \iota }\right),\\ \left(\left(y,z\right),\left[0.58,0.67\right]e^{\left[0.61,0.72\right]2\pi \iota },\left[0.58,0.64\right]e^{\left[0.64.0.75\right]2\pi \iota },\left[0.62,0.69\right]e^{\left[0.71,0.78\right]2\pi \iota }\right),\\ \left(\left(z,x\right),\left[0.58,0.70\right]e^{\left[0.61,0.71\right]2\pi \iota },\left[0.61,0.68\right]e^{\left[0.64.0.73\right]2\pi \iota },\left[0.69,0.72\right]e^{\left[0.61,0.75\right]2\pi \iota }\right),\\ \left(\left(z,y\right),\left[0.58,0.67\right]e^{\left[0.61,0.72\right]2\pi \iota },\left[0.58,0.64\right]e^{\left[0.64.0.75\right]2\pi \iota },\left[0.62,0.69\right]e^{\left[0.71,0.78\right]2\pi \iota }\right),\\ \left(\left(z,z\right),\left[0.58,0.73\right]e^{\left[0.61,0.78\right]2\pi \iota },\left[0.61,0.87\right]e^{\left[0.64.0.84\right]2\pi \iota },\left[0.59,0.65\right]e^{\left[0.61,0.75\right]2\pi \iota }\right) \end{array}\right\}\text{then.} \end{array}$$

(1) The IVCT-spherical preorder-FR R_1_ on *Ӻ* is
  (56) $$\begin{array}{c} {\text{R}}_{1}=\left\{\begin{array}{c} \left(\left(x,x\right),\left[0.64,0.70\right]e^{\left[0.65,0.71\right]2\pi \iota },\left[0.66,0.68\right]e^{\left[0.68.0.73\right]2\pi \iota },\left[0.69,0.72\right]e^{\left[0.61,0.74\right]2\pi \iota }\right),\\ \left(\left(y,x\right),\left[0.62,0.67\right]e^{\left[0.65,0.71\right]2\pi \iota },\left[0.58,0.64\right]e^{\left[0.68.0.73\right]2\pi \iota },\left[0.69,0.72\right]e^{\left[0.71,0.78\right]2\pi \iota }\right),\\ \left(\left(y,y\right),\left[0.62,0.67\right]e^{\left[0.68,0.72\right]2\pi \iota },\left[0.58,0.64\right]e^{\left[0.68.0.75\right]2\pi \iota },\left[0.62,0.69\right]e^{\left[0.71,0.78\right]2\pi \iota }\right),\\ \left(\left(z,z\right),\left[0.58,0.73\right]e^{\left[0.61,0.78\right]2\pi \iota },\left[0.61,0.87\right]e^{\left[0.64.0.84\right]2\pi \iota },\left[0.59,0.65\right]e^{\left[0.61,0.75\right]2\pi \iota }\right) \end{array}\right\}. \end{array}$$
(2) The IVCT-spherical strict order-FR R_2_ on *Ӻ* is
  (57) $$\begin{array}{c} {\text{R}}_{2}=\left\{\begin{array}{c} \left(\left(y,x\right),\left[0.62,0.67\right]e^{\left[0.65,0.71\right]2\pi \iota },\left[0.58,0.64\right]e^{\left[0.68.0.73\right]2\pi \iota },\left[0.69,0.72\right]e^{\left[0.71,0.78\right]2\pi \iota }\right),\\ \left(\left(z,x\right),\left[0.58,0.70\right]e^{\left[0.61,0.71\right]2\pi \iota },\left[0.61,0.68\right]e^{\left[0.64.0.73\right]2\pi \iota },\left[0.69,0.72\right]e^{\left[0.61,0.75\right]2\pi \iota }\right),\\ \left(\left(z,y\right),\left[0.58,0.67\right]e^{\left[0.61,0.72\right]2\pi \iota },\left[0.58,0.64\right]e^{\left[0.64.0.75\right]2\pi \iota },\left[0.62,0.69\right]e^{\left[0.71,0.78\right]2\pi \iota }\right) \end{array}\right\}. \end{array}$$
(3) The IVCT-spherical linear order-FR R_3_ on *Ӻ* is
  (58) $$\begin{array}{c} {\text{R}}_{3}=\left\{\begin{array}{c} \left(\left(x,x\right),\left[0.64,0.70\right]e^{\left[0.65,0.71\right]2\pi \iota },\left[0.66,0.68\right]e^{\left[0.68.0.73\right]2\pi \iota },\left[0.69,0.72\right]e^{\left[0.61,0.74\right]2\pi \iota }\right),\\ \left(\left(y,x\right),\left[0.62,0.67\right]e^{\left[0.65,0.71\right]2\pi \iota },\left[0.58,0.64\right]e^{\left[0.68.0.73\right]2\pi \iota },\left[0.69,0.72\right]e^{\left[0.71,0.78\right]2\pi \iota }\right),\\ \left(\left(y,y\right),\left[0.62,0.67\right]e^{\left[0.68,0.72\right]2\pi \iota },\left[0.58,0.64\right]e^{\left[0.68.0.75\right]2\pi \iota },\left[0.62,0.69\right]e^{\left[0.71,0.78\right]2\pi \iota }\right),\\ \left(\left(z,x\right),\left[0.58,0.70\right]e^{\left[0.61,0.71\right]2\pi \iota },\left[0.61,0.68\right]e^{\left[0.64.0.73\right]2\pi \iota },\left[0.69,0.72\right]e^{\left[0.61,0.75\right]2\pi \iota }\right),\\ \left(\left(z,y\right),\left[0.58,0.67\right]e^{\left[0.61,0.72\right]2\pi \iota },\left[0.58,0.64\right]e^{\left[0.64.0.75\right]2\pi \iota },\left[0.62,0.69\right]e^{\left[0.71,0.78\right]2\pi \iota }\right),\\ \left(\left(z,z\right),\left[0.58,0.73\right]e^{\left[0.61,0.78\right]2\pi \iota },\left[0.61,0.87\right]e^{\left[0.64.0.84\right]2\pi \iota },\left[0.59,0.65\right]e^{\left[0.61,0.75\right]2\pi \iota }\right) \end{array}\right\}. \end{array}$$

The IVCT-spherical partial order-FR R_4_ on *Ӻ* is

(59) $$\begin{array}{c} {\text{R}}_{4}=\left\{\begin{array}{c} \left(\left(x,x\right),\left[0.64,0.70\right]e^{\left[0.65,0.71\right]2\pi \iota },\left[0.66,0.68\right]e^{\left[0.68.0.73\right]2\pi \iota },\left[0.69,0.72\right]e^{\left[0.61,0.74\right]2\pi \iota }\right),\\ \left(\left(y,x\right),\left[0.62,0.67\right]e^{\left[0.65,0.71\right]2\pi \iota },\left[0.58,0.64\right]e^{\left[0.68.0.73\right]2\pi \iota },\left[0.69,0.72\right]e^{\left[0.71,0.78\right]2\pi \iota }\right),\\ \left(\left(y,y\right),\left[0.62,0.67\right]e^{\left[0.68,0.72\right]2\pi \iota },\left[0.58,0.64\right]e^{\left[0.68.0.75\right]2\pi \iota },\left[0.62,0.69\right]e^{\left[0.71,0.78\right]2\pi \iota }\right),\\ \left(\left(z,x\right),\left[0.58,0.70\right]e^{\left[0.61,0.71\right]2\pi \iota },\left[0.61,0.68\right]e^{\left[0.64.0.73\right]2\pi \iota },\left[0.69,0.72\right]e^{\left[0.61,0.75\right]2\pi \iota }\right),\\ \left(\left(z,z\right),\left[0.58,0.73\right]e^{\left[0.61,0.78\right]2\pi \iota },\left[0.61,0.87\right]e^{\left[0.64.0.84\right]2\pi \iota },\left[0.59,0.65\right]e^{\left[0.61,0.75\right]2\pi \iota }\right) \end{array}\right\}. \end{array}$$

**Definition 15 .**

A Hasse diagram (H-diagram) represents the relationship between members of a partially ordered set with an indicated upward direction.

1. A maximal element in an H-diagram that is not less than any other H-diagram element
2. A minimal element in an H-diagram that is not greater than any other H-diagram element
3. A maximum element in an H-diagram that is not less than any other H-diagram element and all other elements related to this element
4. A minimum element in an H-diagram that is not greater than any other H-diagram element and this element related to all other elements

**Example 9 .**

Suppose that *F*={*e*, *f*, *g*, *h*, *i*, *j*, *k*, *l*, *m*, *o*, *n*} is the element of the IVCT-spherical partial order fuzzy set.

We neglect the membership, abstinence, and nonmembership grades just for simplicity.

**Figure 2:**
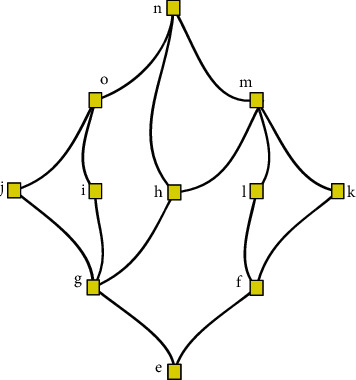
H-diagram for the minimal, maximal, minimum, and maximum elements.

The H-diagram of set is given in Figure 2.

In this H-diagram, we have the following:

1. Maximal and maximum element is *n*
2. Minimal element is *e*
3. Minimum element is *e*

## 4. Applications

In this section, the applications of the theories presented are described. We applied the presented relationships and their many aspects to computer systems, namely, “cybersecurity and cybercrime in industrial control systems.”

### 4.1. Security Measures

The mechanization of industrial systems has progressively gained traction over the last few generations. Since business necessitates continuous improvement in the efficiency of the manufacturing process, industries are connected to the company network and are frequently operated remotely over the network. As a result, (IT) absorption and network connectivity continue to increase. In addition to their advantages, these advanced technologies have accidentally added new threats to the factory automation sector. The Industrial Control Systems that are currently in use were created to last generations, and many of them were created without consideration for (IT) security. A number of security measures are discussed in the following.

#### 4.1.1. Data Backup (DB)

The purpose of the backup is to create a copy of the file that can be recovered if the original data is lost. Data loss can be caused by hardware or software failures, data corruption, or a beings incident, such as “virus or malware” or unintentional deletion of data.

*(1) Cryptography (CG)*. Using an encryption method, cryptography is the process of storing and transferring data and information in such a way that only those who require it may read, access, or analyze it. To protect the data, storage, and user authentication, we use cryptography.

*(2) Antivirus (AV)*. Antivirus software scans for, detects, and removes malware from computers. While antivirus app is installed, it usually runs in the background, giving real-time protection against computer viruses.

*(3) Antispyware (AS)*. Antispyware application is able to detect and remove malicious malware. Spyware is a type of software that is installed on a computer without the user's knowledge in order to collect personal data.

*(4) Firewall (FW)*. A firewall is a network security system that monitors network connections and admits or disallows data packets based on security policies.

*(5) Human Aspects (HA)*. A technique for testing security awareness is the Human Aspects of Data Security Survey.

**Figure 3:**
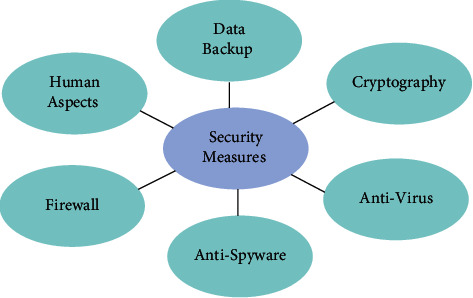
Flow chart for the security measures.

The factors of security measure are depicted in Figure 3.

The security measures listed above are abbreviated and provided degrees of membership, abstinence, and nonmembership values in Table 2.

### 4.2. Sources of Threats

There are many faults in a computer system. Cybercriminals are always on the lookout for new ways to make money. There are several sources of threats.

- Hackers (HK)
- Malicious Insider (MI)
- Nation States (NS)
- Malware (ML)

**Figure 4:**
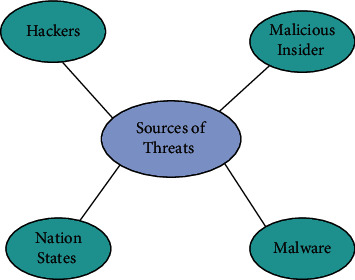
Flow chart for the sources of threats.

Figure 4 shows the factors of sources of threats.

The above sources of threats are abbreviated and provided degrees of membership, abstinence, and nonmembership values in Table 3.

### 4.3. Calculations

The relationships between each cybersecurity approach's effectiveness, neutrality, and lack of effectiveness against each cybercrime are studied. The following calculations are carried out. Let *F* and *E* be two IVCT-spherical-fuzzy sets:

(60) $$\begin{array}{c} F=\left\{\begin{array}{c} \left(\text{DB},\left[0.42,0.48\right]e^{\left[0.44,0.49\right]2\pi \iota },\left[0.49,0.58\right]e^{\left[0.50.0.59\right]2\pi \iota },\left[0.51,0.61\right]e^{\left[0.48,0.63\right]2\pi \iota }\right),\\ \left(\text{CG},\left[0.44,0.51\right]e^{\left[0.49,0.61\right]2\pi \iota },\left[0.47,0.56\right]e^{\left[0.62.0.64\right]2\pi \iota },\left[0.48,0.61\right]e^{\left[0.48,0.67\right]2\pi \iota }\right),\\ \left(\text{AV},\left[0.52,0.54\right]e^{\left[0.55,0.61\right]2\pi \iota },\left[0.52,0.63\right]e^{\left[0.56.0.64\right]2\pi \iota },\left[0.45,0.63\right]e^{\left[0.67,0.69\right]2\pi \iota }\right),\\ \left(\text{AS},\left[0.49,0.58\right]e^{\left[0.64,0.69\right]2\pi \iota },\left[0.53,0.65\right]e^{\left[0.67.0.71\right]2\pi \iota },\left[0.52,0.65\right]e^{\left[0.49,0.67\right]2\pi \iota }\right),\\ \left(\text{FW},\left[0.49,0.56\right]e^{\left[0.56,0.63\right]2\pi \iota },\left[0.49,0.53\right]e^{\left[0.54.0.67\right]2\pi \iota },\left[0.54,0.59\right]e^{\left[0.52,0.64\right]2\pi \iota }\right),\\ \left(\text{HA},\left[0.44,0.58\right]e^{\left[0.64,0.69\right]2\pi \iota },\left[0.63,0.67\right]e^{\left[0.67.0.72\right]2\pi \iota },\left[0.63,0.66\right]e^{\left[0.56,0.68\right]2\pi \iota }\right) \end{array}\right\},\\ \prime \prime E=\left\{\begin{array}{c} \left(\text{HK},\left[0.34,0.45\right]e^{\left[0.45,0.49\right]2\pi \iota },\left[0.37,0.45\right]e^{\left[0.54.0.58\right]2\pi \iota },\left[0.49,0.55\right]e^{\left[0.55,0.59\right]2\pi \iota }\right),\\ \left(\text{MI},\left[0.39,0.55\right]e^{\left[0.55,0.59\right]2\pi \iota },\left[0.43,0.55\right]e^{\left[0.57.0.59\right]2\pi \iota },\left[0.48,0.58\right]e^{\left[0.64,0.67\right]2\pi \iota }\right),\\ \left(\text{NS},\left[0.49,0.57\right]e^{\left[0.56,0.59\right]2\pi \iota },\left[0.53,0.65\right]e^{\left[0.65.0.69\right]2\pi \iota },\left[0.61,0.65\right]e^{\left[0.53,0.59\right]2\pi \iota }\right),\\ \left(\text{ML},\left[0.49,0.59\right]e^{\left[0.65,0.69\right]2\pi \iota },\left[0.38,0.67\right]e^{\left[0.55.0.71\right]2\pi \iota },\left[0.59,0.68\right]e^{\left[0.63,0.70\right]2\pi \iota }\right) \end{array}\right\}. \end{array}$$

We utilize the Cartesian product to determine the efficiency of various security solutions against certain threats. Thus, the Cartesian product between the IVCT-spherical-FSs *F* and *E* is

(61) $$\begin{array}{c} F\times {}^{\prime \prime }E=\left\{\begin{array}{c} \left(\left(\text{DB},\text{HK}\right),\left[0.34,0.45\right]e^{\left[0.44,0.49\right]2\pi \iota },\left[0.37,0.45\right]e^{\left[0.50.0.58\right]2\pi \iota },\left[0.51,0.61\right]e^{\left[0.55,0.63\right]2\pi \iota }\right),\\ \left(\left(\text{DB},\text{MI}\right),\left[0.39,0.48\right]e^{\left[0.44,0.49\right]2\pi \iota },\left[0.43,0.55\right]e^{\left[0.50.0.59\right]2\pi \iota },\left[0.51,0.61\right]e^{\left[0.64,0.67\right]2\pi \iota }\right),\\ \left(\left(\text{DB},\text{NS}\right),\left[0.42,0.48\right]e^{\left[0.44,0.49\right]2\pi \iota },\left[0.49,0.58\right]e^{\left[0.50.0.59\right]2\pi \iota },\left[0.61,0.65\right]e^{\left[0.53,0.63\right]2\pi \iota }\right),\\ \left(\left(\text{DB},\text{ML}\right),\left[0.42,0.48\right]e^{\left[0.44,0.49\right]2\pi \iota },\left[0.38,0.58\right]e^{\left[0.50.0.59\right]2\pi \iota },\left[0.59,0.68\right]e^{\left[0.63,0.70\right]2\pi \iota }\right),\\ \left(\left(\text{CG},\text{HK}\right),\left[0.34,0.45\right]e^{\left[0.45,0.49\right]2\pi \iota },\left[0.37,0.45\right]e^{\left[0.54.0.58\right]2\pi \iota },\left[0.49,0.61\right]e^{\left[0.55,0.67\right]2\pi \iota }\right),\\ \left(\left(\text{CG},\text{MI}\right),\left[0.39,0.51\right]e^{\left[0.49,0.59\right]2\pi \iota },\left[0.43,0.55\right]e^{\left[0.57.0.59\right]2\pi \iota },\left[0.48,0.61\right]e^{\left[0.64,0.67\right]2\pi \iota }\right),\\ \left(\left(\text{CG},\text{NS}\right),\left[0.44,0.51\right]e^{\left[0.49,0.59\right]2\pi \iota },\left[0.47,0.56\right]e^{\left[0.62.0.64\right]2\pi \iota },\left[0.61,0.65\right]e^{\left[0.53,0.67\right]2\pi \iota }\right),\\ \left(\left(\text{CG},\text{ML}\right),\left[0.44,0.51\right]e^{\left[0.49,0.61\right]2\pi \iota },\left[0.38,0.56\right]e^{\left[0.55.0.64\right]2\pi \iota },\left[0.59,0.68\right]e^{\left[0.63,0.70\right]2\pi \iota }\right),\\ \left(\left(\text{AV},\text{HK}\right),\left[0.34,0.45\right]e^{\left[0.45,0.49\right]2\pi \iota },\left[0.37,0.45\right]e^{\left[0.54.0.58\right]2\pi \iota },\left[0.49,0.63\right]e^{\left[0.67,0.69\right]2\pi \iota }\right),\\ \left(\left(\text{AV},\text{MI}\right),\left[0.39,0.54\right]e^{\left[0.55,0.59\right]2\pi \iota },\left[0.43,0.55\right]e^{\left[0.56.0.59\right]2\pi \iota },\left[0.48,0.63\right]e^{\left[0.67,0.69\right]2\pi \iota }\right),\\ \left(\left(\text{AV},\text{NS}\right),\left[0.49,0.54\right]e^{\left[0.56,0.59\right]2\pi \iota },\left[0.52,0.63\right]e^{\left[0.56.0.64\right]2\pi \iota },\left[0.61,0.65\right]e^{\left[0.67,0.69\right]2\pi \iota }\right),\\ \left(\left(\text{AV},\text{ML}\right),\left[0.49,0.58\right]e^{\left[0.64,0.69\right]2\pi \iota },\left[0.38,0.65\right]e^{\left[0.55.0.71\right]2\pi \iota },\left[0.59,0.68\right]e^{\left[0.67,0.70\right]2\pi \iota }\right),\\ \left(\left(\text{AS},\text{HK}\right),\left[0.34,0.45\right]e^{\left[0.45,0.49\right]2\pi \iota },\left[0.37,0.45\right]e^{\left[0.54.0.58\right]2\pi \iota },\left[0.52,0.65\right]e^{\left[0.45,0.67\right]2\pi \iota }\right),\\ \left(\left(\text{AS},\text{MI}\right),\left[0.39,0.55\right]e^{\left[0.55,0.59\right]2\pi \iota },\left[0.43,0.55\right]e^{\left[0.57.0.59\right]2\pi \iota },\left[0.52,0.65\right]e^{\left[0.64,0.67\right]2\pi \iota }\right),\\ \left(\left(\text{AS},\text{NS}\right),\left[0.49,0.57\right]e^{\left[0.56,0.59\right]2\pi \iota },\left[0.53,0.65\right]e^{\left[0.65.0.69\right]2\pi \iota },\left[0.61,0.65\right]e^{\left[0.53,0.67\right]2\pi \iota }\right),\\ \left(\left(\text{AS},\text{ML}\right),\left[0.49,0.58\right]e^{\left[0.64,0.69\right]2\pi \iota },\left[0.38,0.65\right]e^{\left[0.55.0.71\right]2\pi \iota },\left[0.59,0.68\right]e^{\left[0.63,0.70\right]2\pi \iota }\right),\\ \left(\left(\text{FW},\text{HK}\right),\left[0.34,0.45\right]e^{\left[0.45,0.49\right]2\pi \iota },\left[0.37,0.45\right]e^{\left[0.54.0.58\right]2\pi \iota },\left[0.54,0.59\right]e^{\left[0.55,0.64\right]2\pi \iota }\right),\\ \left(\left(\text{FW},\text{MI}\right),\left[0.39,0.55\right]e^{\left[0.55,0.59\right]2\pi \iota },\left[0.43,0.53\right]e^{\left[0.54.0.59\right]2\pi \iota },\left[0.54,0.59\right]e^{\left[0.64,0.67\right]2\pi \iota }\right),\\ \left(\left(\text{FW},\text{NS}\right),\left[0.49,0.56\right]e^{\left[0.56,0.59\right]2\pi \iota },\left[0.49,0.53\right]e^{\left[0.54.0.67\right]2\pi \iota },\left[0.61,0.65\right]e^{\left[0.53,0.64\right]2\pi \iota }\right),\\ \left(\left(\text{FW},\text{ML}\right),\left[0.54,0.59\right]e^{\left[0.56,0.63\right]2\pi \iota },\left[0.38,0.53\right]e^{\left[0.64.0.67\right]2\pi \iota },\left[0.59,0.68\right]e^{\left[0.63,0.70\right]2\pi \iota }\right),\\ \left(\left(\text{HA},\text{HK}\right),\left[0.34,0.45\right]e^{\left[0.45,0.49\right]2\pi \iota },\left[0.37,0.45\right]e^{\left[0.54.0.58\right]2\pi \iota },\left[0.63,0.66\right]e^{\left[0.56,0.68\right]2\pi \iota }\right),\\ \left(\left(\text{HA},\text{MI}\right),\left[0.39,0.55\right]e^{\left[0.55,0.59\right]2\pi \iota },\left[0.43,0.55\right]e^{\left[0.57.0.59\right]2\pi \iota },\left[0.63,0.66\right]e^{\left[0.64,0.68\right]2\pi \iota }\right),\\ \left(\left(\text{HA},\text{NS}\right),\left[0.44,0.57\right]e^{\left[0.56,0.59\right]2\pi \iota },\left[0.53,0.65\right]e^{\left[0.65.0.69\right]2\pi \iota },\left[0.63,0.66\right]e^{\left[0.56,0.68\right]2\pi \iota }\right),\\ \left(\left(\text{HA},\text{ML}\right),\left[0.44,0.58\right]e^{\left[0.64,0.69\right]2\pi \iota },\left[0.38,0.67\right]e^{\left[0.55.0.71\right]2\pi \iota },\left[0.63,0.66\right]e^{\left[0.63,0.70\right]2\pi \iota }\right) \end{array}\right\}. \end{array}$$

Every element of  *F* × *E* is an order pair, which describes the relationship between the two factors in an ordered pair, that is, the influences and effects of the first parameter on the second. The degrees of membership show how efficient a security measure is at overcoming a specific source of risk over time. The degrees of abstinence indicate no effect at all. On the other hand, the degrees of nonmembership indicate the ineffectiveness of a security measure against a particular source of threat information. The ordered pair, for example, ((DB, ML), [0.42, 0.48]*e*^[0.44, 0.49]2*πι*^, [0.38, 0.58]*e*^[0.50.0.59]2*πι*^, [0.59, 0.68]*e*^[0.63, 0.70]2*πι*^), indicates that the backup data option can successfully combat the hazards posed by malware. Moreover, the numbers suggest that inefficiency is at a minimum. The following is a translation of the degree values: the grade of security provided by a data backup method against threats injection via malware is 42 to 48 percent, in terms of 8 to 10 time units, and the possibilities of a cyberattack via malware bypassing the data backup mode are 61 to 65 percent, in terms of 2 to 5 time units. As far as the security is concerned, the longer period of time in the degree of membership is considered better; on the other hand, smaller time frame in the degree of nonmembership is better.

### 4.4. Cybersecurity Best Practices for Business

How can you keep your company from becoming a victim of a cyberattack? Here are eight cybersecurity recommended practices for businesses that you can start using right now.

#### 4.4.1. Use a Firewall (UFW)

A firewall is one of the first points of protection in a cyberattack. To provide additional safety, several companies are beginning to build internal firewalls. Employees who work from home should also have a firewall installed on their home network.

(62) $$\begin{array}{c} \left(\text{UFW},\left[0.34,0.45\right]e^{\left[0.45,0.49\right]2\pi \iota },\left[0.37,0.45\right]e^{\left[0.54.0.58\right]2\pi \iota },\left[0.49,0.55\right]e^{\left[0.55,0.59\right]2\pi \iota }\right). \end{array}$$

#### 4.4.2. Document Your security Policies (DCSP)

While many businesses rely on word of mouth and gut reaction, cybersecurity is one place where it is critical to document your procedures.

(63) $$\begin{array}{c} \left(\text{DCSP},\left[0.42,0.48\right]e^{\left[0.44,0.49\right]2\pi \iota },\left[0.49,0.58\right]e^{\left[0.50.0.59\right]2\pi \iota },\left[0.51,0.61\right]e^{\left[0.48,0.63\right]2\pi \iota }\right). \end{array}$$

#### 4.4.3. Plan for Mobile Devices (PMD)

With the growing popularity of wearables like smart watches and fitness trackers that can connect to the Internet, it is critical to include these devices in a strategy. The PMD plan will have an impact on industrial security.

(64) $$\begin{array}{c} \left(\text{PMD},\left[0.39,0.55\right]e^{\left[0.55,0.59\right]2\pi \iota },\left[0.43,0.55\right]e^{\left[0.57.0.59\right]2\pi \iota },\left[0.48,0.58\right]e^{\left[0.64,0.67\right]2\pi \iota }\right). \end{array}$$

#### 4.4.4. Educate All Employees (EAE)

To keep employees accountable, have each employee sign a statement declaring that they have been aware of the policies and realize that if they do not obey security policies, actions may be taken against them.

(65) $$\begin{array}{c} \left(\text{EAE},\left[0.44,0.58\right]e^{\left[0.64,0.69\right]2\pi \iota },\left[0.63,0.67\right]e^{\left[0.67.0.72\right]2\pi \iota },\left[0.63,0.66\right]e^{\left[0.56,0.68\right]2\pi \iota }\right). \end{array}$$

#### 4.4.5. Enforce Safe Password Practices (ESPP)

Employees dislike changing passwords, but, given the current circumstances, all employee devices connecting to the company network must be password-protected.

(66) $$\begin{array}{c} \left(\text{ESPP},\left[0.49,0.58\right]e^{\left[0.64,0.69\right]2\pi \iota },\left[0.53,0.65\right]e^{\left[0.67.0.71\right]2\pi \iota },\left[0.52,0.65\right]e^{\left[0.49,0.67\right]2\pi \iota }\right). \end{array}$$

#### 4.4.6. Regularly Back Up All Data (RBAD)

While it is essential to prevent as many attacks as possible, no matter how careful you are, you can still get attacked. Ensure that any data you have stored to the cloud is backed up. In the event of a fire or flood, make sure duplicates are kept in a separate area.

(67) $$\begin{array}{c} \left(\text{RBAD},\left[0.45,0.58\right]e^{\left[0.64,0.70\right]2\pi \iota },\left[0.53,0.61\right]e^{\left[0.67.0.69\right]2\pi \iota },\left[0.53,0.64\right]e^{\left[0.47,0.66\right]2\pi \iota }\right). \end{array}$$

#### 4.4.7. Install Antimalware Software (IAMS)

It is easy to think that your staff is aware of the need for not opening phishing emails. However, some staff open phishing emails, requiring the installation of antimalware software.

(68) $$\begin{array}{c} \left(\text{IAMS},\left[0.49,0.57\right]e^{\left[0.56,0.59\right]2\pi \iota },\left[0.53,0.65\right]e^{\left[0.65.0.69\right]2\pi \iota },\left[0.61,0.65\right]e^{\left[0.53,0.59\right]2\pi \iota }\right). \end{array}$$

#### 4.4.8. Use Multifactor Identification (UMI)

The purpose of security is always changing. Every day, cybercriminals improve their skills. To secure your data as much as possible, it is important that each and every staff member prioritize cybersecurity. Also it is the most essential that you keep up with the latest attack techniques and protection technology. It is critical to your company's success.

(69) $$\begin{array}{c} \left(\text{UMI},\left[0.45,0.54\right]e^{\left[0.53,0.57\right]2\pi \iota },\left[0.51,0.68\right]e^{\left[0.64.0.72\right]2\pi \iota },\left[0.59,0.63\right]e^{\left[0.53,0.62\right]2\pi \iota }\right). \end{array}$$

Give each of the security measures a degree of membership, abstinence, or nonmembership and construct an IVCT-spherical-FS *F*.

(70) $$\begin{array}{c} F=\left\{\begin{array}{c} \left(\text{UFW},\left[0.34,0.45\right]e^{\left[0.45,0.49\right]2\pi \iota },\left[0.37,0.45\right]e^{\left[0.54.0.58\right]2\pi \iota },\left[0.49,0.55\right]e^{\left[0.55,0.59\right]2\pi \iota }\right),\\ \left(\text{DCSP},\left[0.42,0.48\right]e^{\left[0.44,0.49\right]2\pi \iota },\left[0.49,0.58\right]e^{\left[0.50.0.59\right]2\pi \iota },\left[0.51,0.61\right]e^{\left[0.48,0.63\right]2\pi \iota }\right),\\ \left(\text{PMD},\left[0.39,0.55\right]e^{\left[0.55,0.59\right]2\pi \iota },\left[0.43,0.55\right]e^{\left[0.57.0.59\right]2\pi \iota },\left[0.48,0.58\right]e^{\left[0.64,0.67\right]2\pi \iota }\right),\\ \left(\text{EAE},\left[0.44,0.58\right]e^{\left[0.64,0.69\right]2\pi \iota },\left[0.63,0.67\right]e^{\left[0.67.0.72\right]2\pi \iota },\left[0.63,0.66\right]e^{\left[0.56,0.68\right]2\pi \iota }\right),\\ \left(\text{ESPP},\left[0.49,0.58\right]e^{\left[0.64,0.69\right]2\pi \iota },\left[0.53,0.65\right]e^{\left[0.67.0.71\right]2\pi \iota },\left[0.52,0.65\right]e^{\left[0.49,0.67\right]2\pi \iota }\right),\\ \left(\text{RBAD},\left[0.45,0.58\right]e^{\left[0.64,0.70\right]2\pi \iota },\left[0.53,0.61\right]e^{\left[0.67.0.69\right]2\pi \iota },\left[0.53,0.64\right]e^{\left[0.47,0.66\right]2\pi \iota }\right),\\ \left(\text{IAMS},\left[0.49,0.57\right]e^{\left[0.56,0.59\right]2\pi \iota },\left[0.53,0.65\right]e^{\left[0.65.0.69\right]2\pi \iota },\left[0.61,0.65\right]e^{\left[0.53,0.59\right]2\pi \iota }\right),\\ \left(\text{UMI},\left[0.45,0.54\right]e^{\left[0.53,0.57\right]2\pi \iota },\left[0.51,0.68\right]e^{\left[0.64.0.72\right]2\pi \iota },\left[0.59,0.63\right]e^{\left[0.53,0.62\right]2\pi \iota }\right) \end{array}\right\}. \end{array}$$

Then, the Cartesian product of  *F* × *F* is given as

(71) $$\begin{array}{c} F\times F=\left\{\begin{array}{c} \left(\left(\text{UFW},\text{UFW}\right),\left[0.34,0.45\right]e^{\left[0.45,0.49\right]2\pi \iota },\left[0.37,0.45\right]e^{\left[0.54.0.58\right]2\pi \iota },\left[0.49,0.55\right]e^{\left[0.55,0.59\right]2\pi \iota }\right),\\ \left(\left(\text{UFW},\text{DCSP}\right),\left[0.34,0.45\right]e^{\left[0.44,0.49\right]2\pi \iota },\left[0.37,0.45\right]e^{\left[0.54.0.58\right]2\pi \iota },\left[0.51,0.61\right]e^{\left[0.55,0.63\right]2\pi \iota }\right),\\ \left(\left(\text{UFW},\text{PMD}\right),\left[0.34,0.45\right]e^{\left[0.45,0.49\right]2\pi \iota },\left[0.37,0.45\right]e^{\left[0.54.0.58\right]2\pi \iota },\left[0.49,0.58\right]e^{\left[0.64,0.67\right]2\pi \iota }\right),\\ \left(\left(\text{UFW},\text{EAE}\right),\left[0.34,0.45\right]e^{\left[0.45,0.49\right]2\pi \iota },\left[0.37,0.45\right]e^{\left[0.54.0.58\right]2\pi \iota },\left[0.63,0.66\right]e^{\left[0.56,0.68\right]2\pi \iota }\right),\\ \left(\left(\text{UFW},\text{ESPP}\right),\left[0.34,0.45\right]e^{\left[0.45,0.49\right]2\pi \iota },\left[0.37,0.45\right]e^{\left[0.54.0.58\right]2\pi \iota },\left[0.52,0.65\right]e^{\left[0.55,0.67\right]2\pi \iota }\right),\\ \left(\left(\text{UFW},\text{RBAD}\right),\left[0.34,0.45\right]e^{\left[0.45,0.49\right]2\pi \iota },\left[0.37,0.45\right]e^{\left[0.54.0.58\right]2\pi \iota },\left[0.53,0.64\right]e^{\left[0.55,0.66\right]2\pi \iota }\right),\\ \left(\left(\text{UFW},\text{IAMS}\right),\left[0.34,0.45\right]e^{\left[0.45,0.49\right]2\pi \iota },\left[0.37,0.45\right]e^{\left[0.54.0.58\right]2\pi \iota },\left[0.61,0.65\right]e^{\left[0.55,0.69\right]2\pi \iota }\right),\\ \left(\left(\text{UFW},\text{UMI}\right),\left[0.34,0.45\right]e^{\left[0.45,0.49\right]2\pi \iota },\left[0.37,0.45\right]e^{\left[0.54.0.58\right]2\pi \iota },\left[0.59,0.63\right]e^{\left[0.55,0.62\right]2\pi \iota }\right),\\ \left(\left(\text{DCSP},\text{UFW}\right),\left[0.34,0.45\right]e^{\left[0.44,0.49\right]2\pi \iota },\left[0.37,0.45\right]e^{\left[0.54.0.58\right]2\pi \iota },\left[0.51,0.61\right]e^{\left[0.55,0.63\right]2\pi \iota }\right),\\ \left(\left(\text{DCSP},\text{DCSP}\right),\left[0.42,0.48\right]e^{\left[0.44,0.49\right]2\pi \iota },\left[0.49,0.58\right]e^{\left[0.50.0.59\right]2\pi \iota },\left[0.51,0.61\right]e^{\left[0.48,0.63\right]2\pi \iota }\right),\\ \left(\left(\text{DCSP},\text{PMD}\right),\left[0.39,0.48\right]e^{\left[0.44,0.49\right]2\pi \iota },\left[0.43,0.55\right]e^{\left[0.50.0.59\right]2\pi \iota },\left[0.51,0.61\right]e^{\left[0.64,0.67\right]2\pi \iota }\right),\\ \left(\left(\text{DCSP},\text{EAE}\right),\left[0.42,0.48\right]e^{\left[0.44,0.49\right]2\pi \iota },\left[0.49,0.58\right]e^{\left[0.50.0.59\right]2\pi \iota },\left[0.63,0.66\right]e^{\left[0.48,0.68\right]2\pi \iota }\right),\\ \left(\left(\text{DCSP},\text{ESPP}\right),\left[0.42,0.48\right]e^{\left[0.44,0.49\right]2\pi \iota },\left[0.49,0.58\right]e^{\left[0.50.0.59\right]2\pi \iota },\left[0.52,0.65\right]e^{\left[0.49,0.67\right]2\pi \iota }\right),\\ \left(\left(\text{DCSP},\text{RBAD}\right),\left[0.42,0.48\right]e^{\left[0.44,0.49\right]2\pi \iota },\left[0.49,0.58\right]e^{\left[0.50.0.59\right]2\pi \iota },\left[0.53,0.64\right]e^{\left[0.48,0.66\right]2\pi \iota }\right),\\ \left(\left(\text{DCSP},\text{IAMS}\right),\left[0.42,0.48\right]e^{\left[0.44,0.49\right]2\pi \iota },\left[0.49,0.58\right]e^{\left[0.50.0.59\right]2\pi \iota },\left[0.61,0.65\right]e^{\left[0.53,0.69\right]2\pi \iota }\right),\\ \left(\left(\text{DCSP},\text{UMI}\right),\left[0.42,0.48\right]e^{\left[0.44,0.49\right]2\pi \iota },\left[0.49,0.58\right]e^{\left[0.50.0.59\right]2\pi \iota },\left[0.59,0.63\right]e^{\left[0.53,0.63\right]2\pi \iota }\right),\\ \left(\left(\text{PMD},\text{UFM}\right),\left[0.34,0.45\right]e^{\left[0.45,0.49\right]2\pi \iota },\left[0.37,0.45\right]e^{\left[0.54.0.58\right]2\pi \iota },\left[0.49,0.58\right]e^{\left[0.64,0.67\right]2\pi \iota }\right),\\ \left(\left(\text{PMD},\text{DSCP}\right),\left[0.39,0.48\right]e^{\left[0.44,0.49\right]2\pi \iota },\left[0.43,0.55\right]e^{\left[0.50.0.59\right]2\pi \iota },\left[0.51,0.61\right]e^{\left[0.64,0.67\right]2\pi \iota }\right),\\ \left(\left(\text{PMD},\text{PMD}\right),\left[0.39,0.55\right]e^{\left[0.55,0.59\right]2\pi \iota },\left[0.43,0.55\right]e^{\left[0.57.0.59\right]2\pi \iota },\left[0.48,0.58\right]e^{\left[0.64,0.67\right]2\pi \iota }\right),\\ \left(\left(\text{PMD},\text{EAE}\right),\left[0.39,0.55\right]e^{\left[0.55,0.59\right]2\pi \iota },\left[0.43,0.55\right]e^{\left[0.57.0.59\right]2\pi \iota },\left[0.63,0.66\right]e^{\left[0.64,0.68\right]2\pi \iota }\right),\\ \left(\left(\text{PMD},\text{ESPP}\right),\left[0.39,0.55\right]e^{\left[0.55,0.59\right]2\pi \iota },\left[0.43,0.55\right]e^{\left[0.57.0.59\right]2\pi \iota },\left[0.52,0.65\right]e^{\left[0.64,0.67\right]2\pi \iota }\right),\\ \left(\left(\text{PMD},\text{RBAD}\right),\left[0.39,0.55\right]e^{\left[0.55,0.59\right]2\pi \iota },\left[0.43,0.55\right]e^{\left[0.57.0.59\right]2\pi \iota },\left[0.53,0.64\right]e^{\left[0.64,0.67\right]2\pi \iota }\right),\\ \left(\left(\text{PMD},\text{IAMS}\right),\left[0.39,0.55\right]e^{\left[0.55,0.59\right]2\pi \iota },\left[0.43,0.55\right]e^{\left[0.57.0.59\right]2\pi \iota },\left[0.61,0.65\right]e^{\left[0.64,0.69\right]2\pi \iota }\right),\\ \left(\left(\text{PMD},\text{UMI}\right),\left[0.39,0.54\right]e^{\left[0.53,0.57\right]2\pi \iota },\left[0.43,0.55\right]e^{\left[0.57.0.59\right]2\pi \iota },\left[0.59,0.63\right]e^{\left[0.64,0.67\right]2\pi \iota }\right),\\ \left(\left(\text{EAE},\text{UFW}\right),\left[0.34,0.45\right]e^{\left[0.45,0.49\right]2\pi \iota },\left[0.37,0.45\right]e^{\left[0.54.0.58\right]2\pi \iota },\left[0.63,0.66\right]e^{\left[0.56,0.68\right]2\pi \iota }\right),\\ \left(\left(\text{EAE},\text{DCSP}\right),\left[0.42,0.48\right]e^{\left[0.44,0.49\right]2\pi \iota },\left[0.49,0.58\right]e^{\left[0.50.0.59\right]2\pi \iota },\left[0.63,0.66\right]e^{\left[0.48,0.68\right]2\pi \iota }\right),\\ \left(\left(\text{EAE},\text{PMD}\right),\left[0.39,0.55\right]e^{\left[0.55,0.59\right]2\pi \iota },\left[0.43,0.55\right]e^{\left[0.57.0.59\right]2\pi \iota },\left[0.63,0.66\right]e^{\left[0.64,0.68\right]2\pi \iota }\right),\\ \left(\left(\text{EAE},\text{EAE}\right),\left[0.44,0.58\right]e^{\left[0.64,0.69\right]2\pi \iota },\left[0.63,0.67\right]e^{\left[0.67.0.72\right]2\pi \iota },\left[0.63,0.66\right]e^{\left[0.56,0.68\right]2\pi \iota }\right),\\ \left(\left(\text{EAE},\text{ESPP}\right),\left[0.44,0.58\right]e^{\left[0.64,0.69\right]2\pi \iota },\left[0.53,0.65\right]e^{\left[0.67.0.71\right]2\pi \iota },\left[0.63,0.66\right]e^{\left[0.56,0.68\right]2\pi \iota }\right),\\ \left(\left(\text{EAE},\text{RBAD}\right),\left[0.44,0.58\right]e^{\left[0.64,0.69\right]2\pi \iota },\left[0.53,0.61\right]e^{\left[0.67.0.69\right]2\pi \iota },\left[0.63,0.66\right]e^{\left[0.56,0.68\right]2\pi \iota }\right),\\ \left(\left(\text{EAE},\text{IAMS}\right),\left[0.44,0.58\right]e^{\left[0.56,0.59\right]2\pi \iota },\left[0.53,0.65\right]e^{\left[0.65.0.69\right]2\pi \iota },\left[0.63,0.66\right]e^{\left[0.56,0.69\right]2\pi \iota }\right),\\ \left(\left(\text{EAE},\text{UMI}\right),\left[0.44,0.54\right]e^{\left[0.53,0.57\right]2\pi \iota },\left[0.51,0.67\right]e^{\left[0.64.0.72\right]2\pi \iota },\left[0.63,0.66\right]e^{\left[0.56,0.68\right]2\pi \iota }\right),\\ \left(\left(\text{ESPP},\text{UFW}\right),\left[0.34,0.45\right]e^{\left[0.45,0.49\right]2\pi \iota },\left[0.37,0.45\right]e^{\left[0.54.0.58\right]2\pi \iota },\left[0.52,0.65\right]e^{\left[0.55,0.67\right]2\pi \iota }\right) \end{array}\right\}. \end{array}$$

*F* × *F* is continued as

(72) $$\begin{array}{c} F\times F=\left\{\begin{array}{c} \left(\left(\text{ESPP},\text{DCSP}\right),\left[0.42,0.48\right]e^{\left[0.44,0.49\right]2\pi \iota },\left[0.49,0.58\right]e^{\left[0.50.0.59\right]2\pi \iota },\left[0.52,0.65\right]e^{\left[0.49,0.67\right]2\pi \iota }\right),\\ \left(\left(\text{ESPP},\text{PMD}\right),\left[0.39,0.55\right]e^{\left[0.55,0.59\right]2\pi \iota },\left[0.43,0.55\right]e^{\left[0.57.0.59\right]2\pi \iota },\left[0.52,0.65\right]e^{\left[0.64,0.67\right]2\pi \iota }\right),\\ \left(\left(\text{ESPP},\text{EAE}\right),\left[0.44,0.58\right]e^{\left[0.64,0.69\right]2\pi \iota },\left[0.53,0.65\right]e^{\left[0.67.0.71\right]2\pi \iota },\left[0.63,0.66\right]e^{\left[0.56,0.68\right]2\pi \iota }\right),\\ \left(\left(\text{ESPP},\text{ESPP}\right),\left[0.49,0.58\right]e^{\left[0.64,0.69\right]2\pi \iota },\left[0.53,0.65\right]e^{\left[0.67.0.71\right]2\pi \iota },\left[0.52,0.65\right]e^{\left[0.49,0.67\right]2\pi \iota }\right),\\ \left(\left(\text{ESPP},\text{RBAD}\right),\left[0.45,0.58\right]e^{\left[0.64,0.69\right]2\pi \iota },\left[0.53,0.61\right]e^{\left[0.67.0.69\right]2\pi \iota },\left[0.53,0.65\right]e^{\left[0.49,0.67\right]2\pi \iota }\right),\\ \left(\left(\text{ESPP},\text{IAMS}\right),\left[0.45,0.58\right]e^{\left[0.64,0.69\right]2\pi \iota },\left[0.53,0.61\right]e^{\left[0.67.0.69\right]2\pi \iota },\left[0.53,0.65\right]e^{\left[0.49,0.67\right]2\pi \iota }\right),\\ \left(\left(\text{ESPP},\text{UMI}\right),\left[0.45,0.54\right]e^{\left[0.53,0.57\right]2\pi \iota },\left[0.51,0.65\right]e^{\left[0.64.0.71\right]2\pi \iota },\left[0.59,0.63\right]e^{\left[0.53,0.67\right]2\pi \iota }\right),\\ \left(\left(\text{RBAD},\text{UFW}\right),\left[0.34,0.45\right]e^{\left[0.45,0.49\right]2\pi \iota },\left[0.37,0.45\right]e^{\left[0.54.0.58\right]2\pi \iota },\left[0.53,0.64\right]e^{\left[0.55,0.66\right]2\pi \iota }\right),\\ \left(\left(\text{RBAD},\text{PMD}\right),\left[0.39,0.55\right]e^{\left[0.55,0.59\right]2\pi \iota },\left[0.43,0.55\right]e^{\left[0.57.0.59\right]2\pi \iota },\left[0.53,0.64\right]e^{\left[0.64,0.67\right]2\pi \iota }\right),\\ \left(\left(\text{RBAD},\text{EAE}\right),\left[0.44,0.58\right]e^{\left[0.64,0.69\right]2\pi \iota },\left[0.53,0.61\right]e^{\left[0.67.0.69\right]2\pi \iota },\left[0.63,0.66\right]e^{\left[0.56,0.68\right]2\pi \iota }\right),\\ \left(\left(\text{RBAD},\text{ESPP}\right),\left[0.45,0.58\right]e^{\left[0.64,0.69\right]2\pi \iota },\left[0.53,0.61\right]e^{\left[0.67.0.69\right]2\pi \iota },\left[0.53,0.65\right]e^{\left[0.49,0.67\right]2\pi \iota }\right),\\ \left(\left(\text{RBAD},\text{RBAD}\right),\left[0.45,0.58\right]e^{\left[0.64,0.70\right]2\pi \iota },\left[0.53,0.61\right]e^{\left[0.67.0.69\right]2\pi \iota },\left[0.53,0.64\right]e^{\left[0.47,0.66\right]2\pi \iota }\right),\\ \left(\left(\text{RBAD},\text{IAMS}\right),\left[0.45,0.57\right]e^{\left[0.56,0.59\right]2\pi \iota },\left[0.53,0.61\right]e^{\left[0.65.0.69\right]2\pi \iota },\left[0.61,0.65\right]e^{\left[0.53,0.69\right]2\pi \iota }\right),\\ \left(\left(\text{RBAD},\text{UMI}\right),\left[0.45,0.54\right]e^{\left[0.53,0.57\right]2\pi \iota },\left[0.51,0.61\right]e^{\left[0.64.0.69\right]2\pi \iota },\left[0.59,0.64\right]e^{\left[0.53,0.66\right]2\pi \iota }\right),\\ \left(\left(\text{IAMS},\text{UFW}\right),\left[0.34,0.45\right]e^{\left[0.45,0.49\right]2\pi \iota },\left[0.37,0.45\right]e^{\left[0.54.0.58\right]2\pi \iota },\left[0.61,0.65\right]e^{\left[0.55,0.69\right]2\pi \iota }\right),\\ \left(\left(IAMS,DC\;SP\right),\left[0.42,0.48\right]e^{\left[0.44,0.49\right]2\pi \iota },\left[0.49,0.58\right]e^{\left[0.50.0.59\right]2\pi \iota },\left[0.61,0.65\right]e^{\left[0.53,0.69\right]2\pi \iota }\right),\\ \left(\left(\text{IAMS},\text{PMD}\right),\left[0.39,0.55\right]e^{\left[0.55,0.59\right]2\pi \iota },\left[0.43,0.55\right]e^{\left[0.57.0.59\right]2\pi \iota },\left[0.61,0.65\right]e^{\left[0.64,0.69\right]2\pi \iota }\right),\\ \left(\left(\text{IAMS},\text{EAE}\right),\left[0.44,0.58\right]e^{\left[0.56,0.59\right]2\pi \iota },\left[0.53,0.65\right]e^{\left[0.65.0.69\right]2\pi \iota },\left[0.63,0.66\right]e^{\left[0.56,0.69\right]2\pi \iota }\right),\\ \left(\left(\text{IAMS},\text{ESPP}\right)\left[0.45,0.58\right]e^{\left[0.64,0.69\right]2\pi \iota },\left[0.53,0.61\right]e^{\left[0.67.0.69\right]2\pi \iota },\left[0.53,0.65\right]e^{\left[0.49,0.67\right]2\pi \iota }\right),\\ \left(\left(\text{IAMS},\text{RBAD}\right),\left[0.45,0.57\right]e^{\left[0.56,0.59\right]2\pi \iota },\left[0.53,0.61\right]e^{\left[0.65.0.69\right]2\pi \iota },\left[0.61,0.65\right]e^{\left[0.53,0.69\right]2\pi \iota }\right),\\ \left(\left(\text{IAMS},\text{IAMS}\right),\left[0.49,0.57\right]e^{\left[0.56,0.59\right]2\pi \iota },\left[0.53,0.65\right]e^{\left[0.65.0.69\right]2\pi \iota },\left[0.61,0.65\right]e^{\left[0.53,0.59\right]2\pi \iota }\right),\\ \left(\left(\text{IAMS},\text{UMI}\right),\left[0.45,0.54\right]e^{\left[0.53,0.57\right]2\pi \iota },\left[0.51,0.65\right]e^{\left[0.64.0.69\right]2\pi \iota },\left[0.61,0.65\right]e^{\left[0.53,0.59\right]2\pi \iota }\right),\\ \left(\left(\text{UMI},\text{UMI}\right),\left[0.45,0.54\right]e^{\left[0.53,0.57\right]2\pi \iota },\left[0.51,0.68\right]e^{\left[0.64.0.72\right]2\pi \iota },\left[0.59,0.63\right]e^{\left[0.53,0.62\right]2\pi \iota }\right),\\ \left(\left(\text{UMI},\text{DCSP}\right),\left[0.42,0.48\right]e^{\left[0.44,0.49\right]2\pi \iota },\left[0.49,0.58\right]e^{\left[0.50.0.59\right]2\pi \iota },\left[0.59,0.63\right]e^{\left[0.53,0.63\right]2\pi \iota }\right),\\ \left(\left(\text{UMI},\text{PMD}\right),\left[0.39,0.54\right]e^{\left[0.53,0.57\right]2\pi \iota },\left[0.43,0.55\right]e^{\left[0.57.0.59\right]2\pi \iota },\left[0.59,0.63\right]e^{\left[0.64,0.67\right]2\pi \iota }\right),\\ \left(\left(\text{UMI},\text{EAE}\right),\left[0.44,0.54\right]e^{\left[0.53,0.57\right]2\pi \iota },\left[0.51,0.67\right]e^{\left[0.64.0.72\right]2\pi \iota },\left[0.63,0.66\right]e^{\left[0.56,0.68\right]2\pi \iota }\right),\\ \left(\left(\text{UMI},\text{ESPP}\right),\left[0.45,0.54\right]e^{\left[0.53,0.57\right]2\pi \iota },\left[0.51,0.65\right]e^{\left[0.64.0.71\right]2\pi \iota },\left[0.59,0.63\right]e^{\left[0.53,0.67\right]2\pi \iota }\right),\\ \left(\left(\text{UMI},\text{RBAD}\right),\left[0.45,0.54\right]e^{\left[0.53,0.57\right]2\pi \iota },\left[0.51,0.61\right]e^{\left[0.64.0.69\right]2\pi \iota },\left[0.59,0.64\right]e^{\left[0.53,0.66\right]2\pi \iota }\right),\\ \left(\left(\text{UMI},\text{IAMS}\right),\left[0.45,0.54\right]e^{\left[0.53,0.57\right]2\pi \iota },\left[0.51,0.65\right]e^{\left[0.64.0.69\right]2\pi \iota },\left[0.61,0.65\right]e^{\left[0.53,0.59\right]2\pi \iota }\right),\\ \left(\left(\text{UMI},\text{UMI}\right),\left[0.45,0.54\right]e^{\left[0.53,0.57\right]2\pi \iota },\left[0.51,0.68\right]e^{\left[0.64.0.72\right]2\pi \iota },\left[0.59,0.63\right]e^{\left[0.53,0.62\right]2\pi \iota }\right) \end{array}\right\}. \end{array}$$

Then the IVCT-spherical partial order-FR R_1_⊆*F* × *F* is given as

(73) $$\begin{array}{c} {\text{R}}_{1}=\left\{\begin{array}{c} \left(\left(\text{UFW},\text{UFW}\right),\left[0.34,0.45\right]e^{\left[0.45,0.49\right]2\pi \iota },\left[0.37,0.45\right]e^{\left[0.54.0.58\right]2\pi \iota },\left[0.49,0.55\right]e^{\left[0.55,0.59\right]2\pi \iota }\right),\\ \left(\left(\text{DCSP},\text{DCSP}\right),\left[0.42,0.48\right]e^{\left[0.44,0.49\right]2\pi \iota },\left[0.49,0.58\right]e^{\left[0.50.0.59\right]2\pi \iota },\left[0.51,0.61\right]e^{\left[0.48,0.63\right]2\pi \iota }\right),\\ \left(\left(\text{PMD},\text{PMD}\right),\left[0.39,0.55\right]e^{\left[0.55,0.59\right]2\pi \iota },\left[0.43,0.55\right]e^{\left[0.57.0.59\right]2\pi \iota },\left[0.48,0.58\right]e^{\left[0.64,0.67\right]2\pi \iota }\right),\\ \left(\left(\text{EAE},\text{EAE}\right),\left[0.44,0.58\right]e^{\left[0.64,0.69\right]2\pi \iota },\left[0.63,0.67\right]e^{\left[0.67.0.72\right]2\pi \iota },\left[0.63,0.66\right]e^{\left[0.56,0.68\right]2\pi \iota }\right),\\ \left(\left(\text{ESPP},\text{ESPP}\right),\left[0.49,0.58\right]e^{\left[0.64,0.69\right]2\pi \iota },\left[0.53,0.65\right]e^{\left[0.67.0.71\right]2\pi \iota },\left[0.52,0.65\right]e^{\left[0.49,0.67\right]2\pi \iota }\right),\\ \left(\left(\text{RBAD},\text{RBAD}\right),\left[0.45,0.58\right]e^{\left[0.64,0.70\right]2\pi \iota },\left[0.53,0.61\right]e^{\left[0.67.0.69\right]2\pi \iota },\left[0.53,0.64\right]e^{\left[0.47,0.66\right]2\pi \iota }\right),\\ \left(\left(\text{IAMS},\text{IAMS}\right),\left[0.49,0.57\right]e^{\left[0.56,0.59\right]2\pi \iota },\left[0.53,0.65\right]e^{\left[0.65.0.69\right]2\pi \iota },\left[0.61,0.65\right]e^{\left[0.53,0.59\right]2\pi \iota }\right),\\ \left(\left(\text{UMI},\text{UMI}\right),\left[0.45,0.54\right]e^{\left[0.53,0.57\right]2\pi \iota },\left[0.51,0.68\right]e^{\left[0.64.0.72\right]2\pi \iota },\left[0.59,0.63\right]e^{\left[0.53,0.62\right]2\pi \iota }\right),\\ \left(\left(\text{UFW},\text{EAE}\right),\left[0.34,0.45\right]e^{\left[0.45,0.49\right]2\pi \iota },\left[0.37,0.45\right]e^{\left[0.54.0.58\right]2\pi \iota },\left[0.63,0.66\right]e^{\left[0.56,0.68\right]2\pi \iota }\right),\\ \left(\left(\text{DCSP},\text{EAE}\right),\left[0.42,0.48\right]e^{\left[0.44,0.49\right]2\pi \iota },\left[0.49,0.58\right]e^{\left[0.50.0.59\right]2\pi \iota },\left[0.63,0.66\right]e^{\left[0.48,0.68\right]2\pi \iota }\right),\\ \left(\left(\text{EAE},\text{RBAD}\right),\left[0.44,0.58\right]e^{\left[0.64,0.69\right]2\pi \iota },\left[0.53,0.61\right]e^{\left[0.67.0.69\right]2\pi \iota },\left[0.63,0.66\right]e^{\left[0.56,0.68\right]2\pi \iota }\right),\\ \left(\left(\text{EAE},\text{PMD}\right),\left[0.39,0.55\right]e^{\left[0.55,0.59\right]2\pi \iota },\left[0.43,0.55\right]e^{\left[0.57.0.59\right]2\pi \iota },\left[0.63,0.66\right]e^{\left[0.64,0.68\right]2\pi \iota }\right),\\ \left(\left(\text{RBAD},\text{ESPP}\right),\left[0.45,0.58\right]e^{\left[0.64,0.69\right]2\pi \iota },\left[0.53,0.61\right]e^{\left[0.67.0.69\right]2\pi \iota },\left[0.53,0.65\right]e^{\left[0.49,0.67\right]2\pi \iota }\right),\\ \left(\left(\text{ESPP},\text{IAMS}\right),\left[0.45,0.58\right]e^{\left[0.64,0.69\right]2\pi \iota },\left[0.53,0.61\right]e^{\left[0.67.0.69\right]2\pi \iota },\left[0.53,0.65\right]e^{\left[0.49,0.67\right]2\pi \iota }\right),\\ \left(\left(\text{PMD},\text{UMI}\right),\left[0.39,0.54\right]e^{\left[0.53,0.57\right]2\pi \iota },\left[0.43,0.55\right]e^{\left[0.57.0.59\right]2\pi \iota },\left[0.59,0.63\right]e^{\left[0.64,0.67\right]2\pi \iota }\right),\\ \left(\left(UMI,IAMS\right),\left[0.45,0.54\right]e^{\left[0.53,0.57\right]2\pi \iota },\left[0.51,0.65\right]e^{\left[0.64.0.69\right]2\pi \iota },\left[0.61,0.65\right]e^{\left[0.53,0.59\right]2\pi \iota }\right),\\ \left(\left(\text{UFW},\text{RBAD}\right),\left[0.34,0.45\right]e^{\left[0.45,0.49\right]2\pi \iota },\left[0.37,0.45\right]e^{\left[0.54.0.58\right]2\pi \iota },\left[0.53,0.64\right]e^{\left[0.55,0.66\right]2\pi \iota }\right),\\ \left(\left(\text{UFW},\text{PMD}\right),\left[0.34,0.45\right]e^{\left[0.45,0.49\right]2\pi \iota },\left[0.37,0.45\right]e^{\left[0.54.0.58\right]2\pi \iota },\left[0.49,0.58\right]e^{\left[0.64,0.67\right]2\pi \iota }\right),\\ \left(\left(\text{DCSP},\text{RBAD}\right),\left[0.42,0.48\right]e^{\left[0.44,0.49\right]2\pi \iota },\left[0.49,0.58\right]e^{\left[0.50.0.59\right]2\pi \iota },\left[0.53,0.64\right]e^{\left[0.48,0.66\right]2\pi \iota }\right),\\ \left(\left(\text{DCSP},\text{PMD}\right),\left[0.39,0.48\right]e^{\left[0.44,0.49\right]2\pi \iota },\left[0.43,0.55\right]e^{\left[0.50.0.59\right]2\pi \iota },\left[0.51,0.61\right]e^{\left[0.64,0.67\right]2\pi \iota }\right),\\ \left(\left(\text{EAE},\text{ESPP}\right),\left[0.44,0.58\right]e^{\left[0.64,0.69\right]2\pi \iota },\left[0.53,0.65\right]e^{\left[0.67.0.71\right]2\pi \iota },\left[0.63,0.66\right]e^{\left[0.56,0.68\right]2\pi \iota }\right),\\ \left(\left(\text{EAE},\text{UMI}\right),\left[0.44,0.54\right]e^{\left[0.53,0.57\right]2\pi \iota },\left[0.51,0.67\right]e^{\left[0.64.0.72\right]2\pi \iota },\left[0.63,0.66\right]e^{\left[0.56,0.68\right]2\pi \iota }\right),\\ \left(\left(\text{RBAD},\text{IAMS}\right),\left[0.45,0.57\right]e^{\left[0.56,0.59\right]2\pi \iota },\left[0.53,0.61\right]e^{\left[0.65.0.69\right]2\pi \iota },\left[0.61,0.65\right]e^{\left[0.53,0.69\right]2\pi \iota }\right),\\ \left(\left(\text{PMD},\text{IAMS}\right),\left[0.39,0.55\right]e^{\left[0.55,0.59\right]2\pi \iota },\left[0.43,0.55\right]e^{\left[0.57.0.59\right]2\pi \iota },\left[0.61,0.65\right]e^{\left[0.64,0.69\right]2\pi \iota }\right) \end{array}\right\}. \end{array}$$

**Figure 5:**
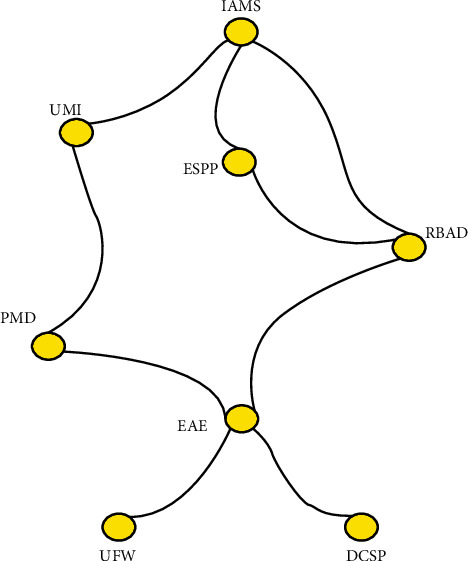
Hasse diagram for IVCT-spherical-partial order *R*_1_.

The Hasse diagram for the given interval-valued complex T-spherical partial order fuzzy relation is shown below. In the following R_1_ diagram, the degrees of membership, abstinence, and nonmembership are concealed for convenience. In Figure 5, it is clearly seen that the strongest security strategy of these eight tools is the IAMS; therefore, it is the maximum as well as the maximal element. On the contrary, the UFM and DCSP are considered to be the minimal element.

**Figure 6:**
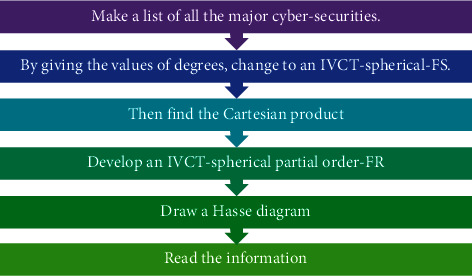
Flow chart of the process discussed in Cybersecurity Best Practices.

The flow chart of the process discussed in above Cybersecurity Best Practices is given in Figure 6.

## 5. Comparative Analysis

A comparison of the proposed methods with existing methods is performed in this section. The IVCT-spherical-FSs and IVCT-spherical-FRs are superior to all other concepts and methods for dealing with fuzziness. These sets clearly discuss three different classes, namely, membership, abstinence, and nonmembership. On the other hand, FSs, CFSs, IVCFSs, IFSs, CIFSs, and IVCIFSs fail. The comparison with IVCPFSs dealing with the applications is given in Table 4.

The comparison with IVC-spherical-FSs dealing with the applications is given in Table 5.

We discussed the supportive, neutral, and discouraging effects of one element on the other in the proposed applications, which were represented by membership, abstinence, and nonmembership grades, accordingly. The abstinence grade must be considered when discussing the overall strength of the effects. Furthermore, IVCPFSs and IVC-spherical-FSs can state all three grades, but they have significant flaws. Table 5 clearly demonstrates that IVCPFSs totally fail to address the problem, since the sum of the grades falls outside of their limits. Although IVC-spherical-FSs have a wider range than IVCPFSs, they barely passed only two times in Table 5. Furthermore, IVC-spherical-FSs were unable to solve the problem since the sum of squares of real and imaginary portions did not fall within the unit interval. Henceforth, considering the dominance of IVCT-spherical-FSs, we used the concept with broader range. IVCT-spherical-FSs allow experts to demonstrate their discernment without limitations.

The properties of different structures in the fuzzy set theory are described in Table 6. The superiority of the IVCT-spherical-FRs structure is shown. It fulfills all four requirements, while other competitors' structures are limited.

## 6. Conclusion

This research established IVCT-spherical-FRs and their various kinds, including IVCT-spherical equivalence-FRs, IVCT-spherical-partial order FRs, IVCT-spherical composite FRs, and IVCT-spherical equivalence-classes. A definition of the Cartesian product of two IVCT-spherical-FSs is also included. The properties of IVCT-spherical-FRs are discussed, as well as some of their interesting outcomes. The Hasse diagram has also been constructed for the IVCT-spherical partial order-FR and IVCT-spherical partial order-FS. Many ideas and concepts related to the Hasse diagram have also been interpreted. For each of the definitions, appropriate examples are provided, and some of the outcomes are demonstrated for the various types of IVCT-spherical-FRs. Additionally, the presented concepts are used to study the relationships between various types of cybersecurity and cybercrime, as well as their sources. By comparing IVCT-spherical-FRs to other mathematical procedures, the section entitled Comparative Analysis confirms the power of IVCT-spherical-FRs. These notions can be extended to further generalizations of FSs in the future, resulting in some very interesting structures that might be employed in a variety of decision-making processes, including group decision-making and multicriteria decision-making.

## Figures and Tables

**Table 1:** The IVCT-spherical-FR R_1_ between the IVCT-spherical-FSs *E* and  *F*.

(*x*, *s*)	[0.51, 0.59]**e**^[0.60, 0.71]2***πι***^, [0.41, 0.52]**e**^[0.45, 0.49]2***πι***^, [0.59, 0.67]**e**^[0.61, 0.69]2***πι***^
(*y*, *r*)	[0.46, 0.53]*e*^[0.59, 0.61]2*πι*^, [0.42, 0.54]*e*^[0.43, 0.47]2*πι*^, [0.61, 0.71]*e*^[0.64, 0.72]2*πι*^
(*y*, *t*)	[0.54, 0.57]*e*^[0.61, 0.72]2*πι*^, [0.42, 0.54]*e*^[0.63, 0.73]2*πι*^, [0.83, 0.90]*e*^[0.64, 0.69]2*πι*^
(*z*, *r*)	[0.46, 0.53]*e*^[0.59, 0.61]2*πι*^, [0.51, 0.56]*e*^[0.43, 0.47]2*πι*^, [0.84, 0.91]*e*^[0.64, 0.69]2*πι*^
(*z*, *t*)	[0.79, 0.81]*e*^[0.61, 0.71]2*πι*^, [0.69, 0.83]*e*^[0.63, 0.73]2*πι*^, [0.84, 0.91]*e*^[0.64, 0.69]2*πι*^

**Table 2:** Details of security measures.

Security measures	Abbreviations	Membership	Abstinence	Nonmembership
Data backup	DB	[0.42, 0.48]e^[0.44, 0.49]2*πι*^	[0.49, 0.58]e^[0.50, 0.59]2*πι*^	[0.51, 0.61]e^[0.48, 0.63]2*πι*^
Cryptography	CG	[0.44, 0.51]e^[0.49, 0.61]2*πι*^	[0.47, 0.56]e^[0.62, 0.64]2*πι*^	[0.48, 0.61]e^[0.48, 0.67]2*πι*^
Antivirus	AV	[0.52, 0.54]e^[0.55, 0.61]2*πι*^	[0.52, 0.63]e^[0.56, 0.64]2*πι*^	[0.45, 0.63]e^[0.67, 0.69]2*πι*^
Antispyware	AS	[0.49, 0.58]e^[0.64, 0.69]2*πι*^	[0.53, 0.65]e^[0.67, 0.71]2*πι*^	[0.52, 0.65]e^[0.49, 0.67]2*πι*^
Firewall	FW	[0.49, 0.56]e^[0.56, 0.63]2*πι*^	[0.49, 0.53]e^[0.54, 0.67]2*πι*^	[0.54, 0.59]e^[0.52, 0.64]2*πι*^
Human Aspects	HA	[0.44, 0.58]e^[0.64, 0.69]2*πι*^	[0.63, 0.67]e^[0.67, 0.72]2*πι*^	[0.63, 0.66]e^[0.56, 0.68]2*πι*^

**Table 3:** Details of sources of threats.

Sources of threats	Abbreviations	Membership	Abstinence	Nonmembership
Hackers	HK	[0.34, 0.45]*e*^[0.45, 0.49]2*πι*^	[0.37, 0.45]*e*^[0.54, 0.58]2*πι*^	[0.49, 0.55]*e*^[0.55, 0.59]2*πι*^
Malicious Insider	MI	[0.39, 0.55]*e*^[0.55, 0.59]2*πι*^	[0.43, 0.55]*e*^[0.57, 0.59]2*πι*^	[0.48, 0.58]*e*^[0.64, 0.67]2*πι*^
Nation States	NS	[0.49, 0.57]*e*^[0.56, 0.59]2*πι*^	[0.53, 0.65]*e*^[0.65, 0.69]2*πι*^	[0.61, 0.65]*e*^[0.53, 0.59]2*πι*^
Malware	ML	[0.49, 0.59]*e*^[0.65, 0.69]2*πι*^	[0.38, 0.67]*e*^[0.55, 0.71]2*πι*^	[0.59, 0.68]*e*^[0.63, 0.70]2*πι*^

**Table 4:** Comparison with IVCPFSs dealing with the applications.

Element	IVCPFS	Result	Status
$\left(\begin{array}{c} \text{UFW},\left[0.34,0.45\right]e^{\left[0.45,0.49\right]2\pi \iota },\\ \left[0.37,0.45\right]e^{\left[0.54.0.58\right]2\pi \iota },\\ \left[0.49,0.55\right]e^{\left[0.55,0.59\right]2\pi \iota } \end{array}\right)$	$\left(\begin{array}{c} 0.45\\ +0.45\\ +0.55 \end{array}\right)e^{\left(\begin{array}{c} 0.49\\ +0.58\\ +0.59 \end{array}\right)}$	1.45*e*^1.66^	Fail
$\left(\begin{array}{c} \text{DCSP},\left[0.42,0.48\right]e^{\left[0.44,0.49\right]2\pi \iota },\\ \left[0.49,0.58\right]e^{\left[0.50.0.59\right]2\pi \iota },\\ \left[0.51,0.61\right]e^{\left[0.48,0.63\right]2\pi \iota } \end{array}\right)$	$\left(\begin{array}{c} 0.48\\ +0.58\\ +0.61 \end{array}\right)e^{\left(\begin{array}{c} 0.49\\ +0.59\\ +0.63 \end{array}\right)}$	1.67*e*^1.81^	Fail
$\left(\begin{array}{c} \text{PMD},\left[0.39,0.55\right]e^{\left[0.55,0.59\right]2\pi \iota },\\ \left[0.43,0.55\right]e^{\left[0.57.0.59\right]2\pi \iota },\\ \left[0.48,0.58\right]e^{\left[0.64,0.67\right]2\pi \iota } \end{array}\right)$	$\left(\begin{array}{c} 0.55\\ +0.55\\ +0.58 \end{array}\right)e^{\left(\begin{array}{c} 0.59\\ +0.59\\ +0.67 \end{array}\right)}$	1.63*e*^1.85^	Fail
$\left(\begin{array}{c} \text{EAE},\left[0.44,0.58\right]e^{\left[0.64,0.69\right]2\pi \iota },\\ \left[0.63,0.67\right]e^{\left[0.67.0.72\right]2\pi \iota },\\ \left[0.63,0.66\right]e^{\left[0.56,0.68\right]2\pi \iota } \end{array}\right)$	$\left(\begin{array}{c} 0.58\\ +0.67\\ +0.66 \end{array}\right)e^{\left(\begin{array}{c} 0.69\\ +0.72\\ +0.68 \end{array}\right)}$	1.91*e*^2.09^	Fail
$\left(\begin{array}{c} \text{ESPP},\left[0.49,0.58\right]e^{\left[0.64,0.69\right]2\pi \iota },\\ \left[0.53,0.65\right]e^{\left[0.67.0.71\right]2\pi \iota },\\ \left[0.52,0.65\right]e^{\left[0.49,0.67\right]2\pi \iota } \end{array}\right)$	$\left(\begin{array}{c} 0.58\\ +0.65\\ +0.65 \end{array}\right)e^{\left(\begin{array}{c} 0.69\\ +0.71\\ +0.67 \end{array}\right)}$	1.88*e*^2.07^	Fail
$\left(\begin{array}{c} \text{RBAD},\left[0.45,0.58\right]e^{\left[0.64,0.70\right]2\pi \iota },\\ \left[0.53,0.61\right]e^{\left[0.67.0.69\right]2\pi \iota },\\ \left[0.53,0.64\right]e^{\left[0.47,0.66\right]2\pi \iota } \end{array}\right)$	$\left(\begin{array}{c} 0.58\\ +0.61\\ +0.64 \end{array}\right)e^{\left(\begin{array}{c} 0.70\\ +0.69\\ +0.66 \end{array}\right)}$	1.83*e*^2.05^	Fail
$\left(\begin{array}{c} \text{IAMS},\left[0.49,0.57\right]e^{\left[0.56,0.59\right]2\pi \iota },\\ \left[0.53,0.65\right]e^{\left[0.65.0.69\right]2\pi \iota },\\ \left[0.61,0.65\right]e^{\left[0.53,0.59\right]2\pi \iota } \end{array}\right)$	$\left(\begin{array}{c} 0.57\\ +0.65\\ +0.65 \end{array}\right)e^{\left(\begin{array}{c} 0.59\\ +0.69\\ +0.59 \end{array}\right)}$	1.87*e*^1.87^	Fail
$\left(\begin{array}{c} \text{UMI},\left[0.45,0.54\right]e^{\left[0.53,0.57\right]2\pi \iota },\\ \left[0.51,0.68\right]e^{\left[0.64.0.72\right]2\pi \iota },\\ \left[0.59,0.63\right]e^{\left[0.53,0.62\right]2\pi \iota } \end{array}\right)$	$\left(\begin{array}{c} 0.54\\ +0.68\\ +0.63 \end{array}\right)e^{\left(\begin{array}{c} 0.57\\ +0.72\\ +0.62 \end{array}\right)}$	1.85*e*^1.91^	Fail

**Table 5:** Comparison with IVC-spherical-FSs dealing with the applications.

Element	IVC-spherical-FS	Result	Status
$\left(\begin{array}{c} \text{UFW},\left[0.34,0.45\right]e^{\left[0.45,0.49\right]2\pi \iota },\\ \left[0.37,0.45\right]e^{\left[0.54.0.58\right]2\pi \iota },\\ \left[0.49,0.55\right]e^{\left[0.55,0.59\right]2\pi \iota } \end{array}\right)$	$\left(\begin{array}{c} {\left(0.45\right)}^{2}\\ +{\left(0.45\right)}^{2}\\ +{\left(0.55\right)}^{2} \end{array}\right)e^{\left(\begin{array}{c} {\left(0.49\right)}^{2}\\ +{\left(0.58\right)}^{2}\\ +{\left(0.59\right)}^{2} \end{array}\right)}$	0.70*e*^0.88^	Pass
$\left(\begin{array}{c} \text{DCSP},\left[0.42,0.48\right]e^{\left[0.44,0.49\right]2\pi \iota },\\ \left[0.49,0.58\right]e^{\left[0.50.0.59\right]2\pi \iota },\\ \left[0.51,0.61\right]e^{\left[0.48,0.63\right]2\pi \iota } \end{array}\right)$	$\left(\begin{array}{c} {\left(0.48\right)}^{2}\\ +{\left(0.58\right)}^{2}\\ +{\left(0.61\right)}^{2} \end{array}\right)e^{\left(\begin{array}{c} {\left(0.49\right)}^{2}\\ +{\left(0.59\right)}^{2}\\ +{\left(0.63\right)}^{2} \end{array}\right)}$	0.93*e*^0.98^	Pass
$\left(\begin{array}{c} \text{PMD},\left[0.39,0.55\right]e^{\left[0.55,0.59\right]2\pi \iota },\\ \left[0.43,0.55\right]e^{\left[0.57.0.59\right]2\pi \iota },\\ \left[0.48,0.58\right]e^{\left[0.64,0.67\right]2\pi \iota } \end{array}\right)$	$\left(\begin{array}{c} {\left(0.55\right)}^{2}\\ +{\left(0.55\right)}^{2}\\ +{\left(0.58\right)}^{2} \end{array}\right)e^{\left(\begin{array}{c} {\left(0.59\right)}^{2}\\ +{\left(0.59\right)}^{2}\\ +{\left(0.67\right)}^{2} \end{array}\right)}$	0.93*e*^1.15^	Fail
$\left(\begin{array}{c} \text{EAE},\left[0.44,0.58\right]e^{\left[0.64,0.69\right]2\pi \iota },\\ \left[0.63,0.67\right]e^{\left[0.67.0.72\right]2\pi \iota },\\ \left[0.63,0.66\right]e^{\left[0.56,0.68\right]2\pi \iota } \end{array}\right)$	$\left(\begin{array}{c} {\left(0.58\right)}^{2}\\ +{\left(0.67\right)}^{2}\\ +{\left(0.66\right)}^{2} \end{array}\right)e^{\left(\begin{array}{c} {\left(0.69\right)}^{2}\\ +{\left(0.72\right)}^{2}\\ +{\left(0.68\right)}^{2} \end{array}\right)}$	1.22*e*^1.44^	Fail
$\left(\begin{array}{c} \text{ESPP},\left[0.49,0.58\right]e^{\left[0.64,0.69\right]2\pi \iota },\\ \left[0.53,0.65\right]e^{\left[0.67.0.71\right]2\pi \iota },\\ \left[0.52,0.65\right]e^{\left[0.49,0.67\right]2\pi \iota } \end{array}\right)$	$\left(\begin{array}{c} {\left(0.58\right)}^{2}\\ +{\left(0.65\right)}^{2}\\ +{\left(0.65\right)}^{2} \end{array}\right)e^{\left(\begin{array}{c} {\left(0.69\right)}^{2}\\ +{\left(0.71\right)}^{2}\\ +{\left(0.67\right)}^{2} \end{array}\right)}$	1.17*e*^1.42^	Fail
$\left(\begin{array}{c} \text{RBAD},\left[0.45,0.58\right]e^{\left[0.64,0.70\right]2\pi \iota },\\ \left[0.53,0.61\right]e^{\left[0.67.0.69\right]2\pi \iota },\\ \left[0.53,0.64\right]e^{\left[0.47,0.66\right]2\pi \iota } \end{array}\right)$	$\left(\begin{array}{c} {\left(0.58\right)}^{2}\\ +{\left(0.61\right)}^{2}\\ +{\left(0.64\right)}^{2} \end{array}\right)e^{\left(\begin{array}{c} {\left(0.70\right)}^{2}\\ +{\left(0.69\right)}^{2}\\ +{\left(0.66\right)}^{2} \end{array}\right)}$	1.08*e*^1.40^	Fail
$\left(\begin{array}{c} \text{IAMS},\left[0.49,0.57\right]e^{\left[0.56,0.59\right]2\pi \iota },\\ \left[0.53,0.65\right]e^{\left[0.65.0.69\right]2\pi \iota },\\ \left[0.61,0.65\right]e^{\left[0.53,0.59\right]2\pi \iota } \end{array}\right)$	$\left(\begin{array}{c} {\left(0.57\right)}^{2}\\ +{\left(0.65\right)}^{2}\\ +{\left(0.65\right)}^{2} \end{array}\right)e^{\left(\begin{array}{c} {\left(0.59\right)}^{2}\\ +{\left(0.69\right)}^{2}\\ +{\left(0.59\right)}^{2} \end{array}\right)}$	1.16*e*^1.15^	Fail
$\left(\begin{array}{c} \text{UMI},\left[0.45,0.54\right]e^{\left[0.53,0.57\right]2\pi \iota },\\ \left[0.51,0.68\right]e^{\left[0.64.0.72\right]2\pi \iota },\\ \left[0.59,0.63\right]e^{\left[0.53,0.62\right]2\pi \iota } \end{array}\right)$	$\left(\begin{array}{c} {\left(0.54\right)}^{2}\\ +{\left(0.68\right)}^{2}\\ +{\left(0.63\right)}^{2} \end{array}\right)e^{\left(\begin{array}{c} {\left(0.57\right)}^{2}\\ +{\left(0.72\right)}^{2}\\ +{\left(0.62\right)}^{2} \end{array}\right)}$	1.14*e*^1.21^	Fail

**Table 6:** Comparison on the basis of structural properties.

Elements	Membership	Abstinence	Nonmembership	Multidimensional
FR	Yes	No	No	No
CFR	Yes	No	No	Yes
IFR	Yes	No	Yes	No
CIFR	Yes	No	Yes	Yes
IVIFR	Yes	Yes	Yes	No
IVCT-spherical-FRs	Yes	Yes	Yes	Yes

## Data Availability

The data used to support the findings of this study are available from the corresponding author upon request.
